# A weighted quantile sum regression with penalized weights and two indices

**DOI:** 10.3389/fpubh.2023.1151821

**Published:** 2023-07-18

**Authors:** Stefano Renzetti, Chris Gennings, Stefano Calza

**Affiliations:** ^1^Department of Medical and Surgical Specialties, Radiological Sciences and Public Health, Università degli Studi di Brescia, Brescia, Italy; ^2^Department of Environmental Medicine and Public Health, Icahn School of Medicine at Mount Sinai, New York, NY, United States; ^3^Department of Molecular and Translational Medicine, Università degli Studi di Brescia, Brescia, Italy

**Keywords:** environmental mixture, weighted quantile sum regression, two indices, penalized weights, nutrients, obesity

## Abstract

**Background:**

New statistical methodologies were developed in the last decade to face the challenges of estimating the effects of exposure to multiple chemicals. Weighted Quantile Sum (WQS) regression is a recent statistical method that allows estimating a mixture effect associated with a specific health effect and identifying the components that characterize the mixture effect.

**Objectives:**

In this study, we propose an extension of WQS regression that estimates two mixture effects of chemicals on a health outcome in the same model through the inclusion of two indices, one in the positive direction and one in the negative direction, with the introduction of a penalization term.

**Methods:**

To evaluate the performance of this new model we performed both a simulation study and a real case study where we assessed the effects of nutrients on obesity among adults using the National Health and Nutrition Examination Survey (NHANES) data.

**Results:**

The method showed good performance in estimating both the regression parameter and the weights associated with the single elements when the penalized term was set equal to the magnitude of the Akaike information criterion of the unpenalized WQS regression. The two indices further helped to give a better estimate of the parameters [Positive direction Median Error (PME): 0.022; Negative direction Median Error (NME): −0.044] compared to the standard WQS without the penalization term (PME: −0.227; NME: 0.215). In the case study, WQS with two indices was able to find a significant effect of nutrients on obesity in both directions identifying sodium and magnesium as the main actors in the positive and negative association, respectively.

**Discussion:**

Through this work, we introduced an extension of WQS regression that improved the accuracy of the parameter estimates when considering a mixture of elements that can have both a protective and a harmful effect on the outcome; and the advantage of adding a penalization term when estimating the weights.

## Introduction

Humans are exposed to many chemicals from multiple chemical classes, based on biomonitoring data, which may influence a particular disease or health state (1–3). Of particular concern is that many of these exposures may act jointly (e.g., along a common adverse pathway) so that even low levels of each may together have an adverse effect. Not accounting for this *mixture effect* may underestimate the potential health risk. Analogously, the food we eat includes many important nutrients. Evaluating dietary quality based on a single nutrient is not adequate.

Several new statistical methodologies were developed to face the challenges of estimating the effects of exposure to multiple chemicals, each addressing its own research question (4). These new methods were developed to solve problems like multiple comparisons and multicollinearity that are commonly encountered with high dimensional and correlated exposures. When using classical statistical models, an increased probability of incurring false positive or false negative results can occur in the first case (5–9) while multicollinearity can produce unstable and biased parameter and standard error estimates in the second case (3, 4, 10–18).

Weighted Quantile Sum (WQS) regression is a recent statistical method that is increasingly applied in epidemiological studies to address the research questions of (i) is there a mixture effect associated with a specific developmental or health effect; and (ii) which of the measured components characterize the mixture effect. This method builds an empirically weighted index that represents the mixture effect in an ensemble first step and tests its association with the outcome of interest in a second step. This body burden index reduces the dimensionality and is more robust to multicollinearity (19, 20). The original methodology provides estimation of a single empirically weighted index that measures the association between the mixture and the dependent variable in only one direction (either positive or negative), which may be interpreted as the joint action of the components in that direction (i.e., a mixture effect). On the other hand, other methods (e.g., variable selection methods) focus on parsimony in predicting an outcome and thus address different research questions. Further, shrinkage methods suffer from limitations in the presence of highly correlated variables like the grouping effect in the elastic net and the arbitrary selection of variables in the LASSO that can be problematic in the risk evaluation of environmental mixtures (19). The estimation of a single index can be an advantage when the elements in the mixture have the same direction in the association with the dependent variable as it focuses inference in the important direction, as is the case when evaluating a mixture effect of jointly acting components. In fact, looking in one direction we avoid the reversal paradox (18) and we increase the power to detect a mixture effect using a single degree of freedom test. However, when the mixture is made of both “good” and “bad” actors (i.e., two sets of components where one set is related to a positive direction and opposite for the other set), it can become a limitation when we want to estimate both the positive mixture effect and negative mixture effect on the specific outcome of interest in a single analysis.

In this study, we propose an extension of WQS regression where two indices are estimated [termed two-indices WQS (2iWQS) in the sequel], one in the positive and the other in the negative direction, in the same model both at the nonlinear estimation ensemble step where the weights are determined and at the final model inference step. This will allow us to use a data reduction approach trying to focus the inference for joint action in now both directions still based on the idea of a mixture effect with a single degree of freedom test for each direction. The simultaneous estimation of the two indices is made possible through the addition of a penalization term when estimating the weights. An application of the new method uses National Health and Nutrition Examination Survey (NHANES) (2011–2016) dietary data and association with obesity. A simulation study is also presented to evaluate the performance of the method.

## Methods

The general formula for the WQS generalized nonlinear regression model for *c* components in the mixture is the following:


$$g\left(\mu \right)=\beta_{0}+\beta_{1}\left(\overset{c}{\underset{i=1}{\sum }}w_{i}q_{i}\right)+z^{\prime }\phi$$

where $w_{i}$ are the unknown weights associated to each component of the mixture to be estimated through an ensemble step using bootstrap samples (19) or random subset samples (21), $q_{i}$ are the values of the components scored into quantiles (quartiles, deciles,…), $\beta_{0}$ is the intercept, $\beta_{1}$ is the coefficient associated to the WQS index, $z^{\prime }\phi$ are the vector of covariates and parameters, respectively and *g*() is any link function between the mean *μ* and the predictor variables as in generalized linear models. WQS regression requires that data are split in a training and validation dataset. The first part of the data is used for the ensemble step to determine the weighted index while accommodating for the correlation among the components, and the final model is fitted on the holdout data to test the significance of $\beta_{1}$ . For the estimated weights the following constraints are imposed: $\sum_{i=1}^{c}w_{i}=1$ ; and $0\leq w_{i}\leq 1$ .

Once the weights are estimated for each ensemble step sample the WQS index is estimated through the formula: $WQS=\sum_{i=1}^{c}{\bar{w}}_{i}q_{i}$ ; where ${\bar{w}}_{i}$ is the mean of the weights found in the ensemble steps associated to either a positive or a negative ${\hat{\beta }}_{1}$ depending on the chosen direction of the association between the mixture and the outcome:


$${\bar{w}}_{i}=\frac{1}{\sum_{b=1}^{B}f\left({\hat{\beta }}_{1\left(b\right)}\right)}\overset{B}{\underset{b=1}{\sum }}w_{i\left(b\right)}f\left({\hat{\beta }}_{1\left(b\right)}\right)$$

where $f\left({\hat{\beta }}_{1\left(b\right)}\right)$ is a signal function defined, for example, as the inverse of the absolute value of the t-test statistic for ${\hat{\beta }}_{1}$ , or $t^{2}$ , or $e^{t}$ (22). The final model is fitted on the holdout validation dataset using the generalized linear model $g\left(\mu \right)=\beta_{0}+\beta_{1}WQS+z^{\prime }\phi$ .

In this study we propose to first include a penalized weight estimate to better identify the truly associated elements in the case of highly correlated data. The objective function (with an identify link) in this case is as follow:


$$\hat{\theta }=\underset{\theta }{\arg\min}\left\{\overset{n}{\underset{j=1}{\sum }}{\left(y_{j}-\left(\beta_{0}+\beta_{1}\left(\overset{c}{\underset{i=1}{\sum }}w_{i}q_{i}\right)+z^{\prime }\phi \right)\right)}^{2}+\lambda \overset{c}{\underset{i=1}{\sum }}\left|v_{i}\right|\right\}$$

where $\theta =\left(\beta_{0},\beta_{1},v_{1},\ldots ,v_{c},\phi ,\lambda \right)$ and where we defined $w_{i}=\frac{v_{i}{}^{2}}{\sum_{i=1}^{c}v_{i}{}^{2}},v_{i}\in ℜ,i=1,\ldots ,c$ to make the penalization term effective.

We further introduce two indices in the same model to allow an estimate of the mixture effect in a positive direction and a mixture effect in a negative direction at the same time, both in the training and validation steps. The new general formula is the following:


$$g\left(\mu \right)=\beta_{0}+\beta_{1p}\left(\overset{c}{\underset{i=1}{\sum }}w_{pi}q_{i}\right)+\beta_{1n}\left(\overset{c}{\underset{i=1}{\sum }}w_{ni}q_{i}\right)+z^{\prime }\phi$$

where $w_{pi}$ and $w_{ni}$ are the unknown weights associated with each component for the positive and negative direction, respectively, while $\beta_{1p}$ and $\beta_{1n}$ are the two parameters that measure the positive and negative effect of the mixture on the outcome. The two indices will be kept in the model both in the first step where two set of weights are estimated (one for the positive and one for the negative direction) and in the second step when the final model is fitted. The equality and inequality constraints are applied to both sets of weights besides a constraint to each $\beta_{1j}$ parameter: $\beta_{1p}\geq 0$ and $\beta_{1n}\leq 0$ . The penalization term is also considered to better discriminate between the elements having an effect and those not associated with the outcome and to reduce the noise produced by the null components that can increase the correlation between the two indices. Without loss of generality, the objective function for the identity link is of the form:


$$\begin{array}{l} \hat{\theta }=\underset{\theta }{\arg\min}\{\overset{n}{\underset{j=1}{\sum }}{\left(y_{j}-\left(\beta_{0}+\beta_{1p}\left(\overset{c}{\underset{i=1}{\sum }}w_{pi}q_{i}\right)+\beta_{1n}\left(\overset{c}{\underset{i=1}{\sum }}w_{ni}q_{i}\right)+z^{\prime }\phi \right)\right)}^{2}\\ +\lambda \left(\overset{c}{\underset{i=1}{\sum }}\left|v_{pi}\right|+\overset{c}{\underset{i=1}{\sum }}\left|v_{ni}\right|\right)\} \end{array}$$

where $\theta =\left(\beta_{0},\beta_{1p},\beta_{1n},v_{p1},\ldots ,v_{pc},v_{n1},\ldots ,v_{nc},\phi ,\lambda \right)$ and $w_{di}=\frac{v_{di}{}^{2}}{\sum_{i=1}^{c}v_{di}{}^{2}},v_{di}\in ℜ,d=p,n,\;i=1,\ldots ,c.$

To set the starting values of the $v_{di}$ we fit two standard WQS regressions constraining the beta in each direction and considering all the observations without splitting the dataset in training and validation and without bootstrapping (for these two starting models we initialize the $v_{di}$ at 1). We then extract the estimated $v_{di}$ from the two models and use them as starting values for the training step of the 2iWQS regression. If one of the two initial WQS regressions does not converge and we are not able to find a set of starting values for the weights in one of the two directions we propose to set the $v_{di}$ equal to the inverse of the elements included in the mixture ($v_{di}=\frac{1}{c}$ ).

The final model for the validation step is $g\left(\mu \right)=\beta_{0}+\beta_{1p}WQS_{p}+\beta_{1n}WQS_{n}+z^{\prime }\phi$ .

To further control the collinearity between the two indices we apply a different signal function when averaging the weights estimated in each ensemble sample in the training step. In particular, we considered the tolerance (the inverse of the variance inflation factor (VIF)) as the weight in the weighted mean: $f\left({\hat{\beta }}_{1d\left(b\right)}\right)={\left(tol\left({\hat{\beta }}_{1d\left(b\right)}\right)/\overset{B}{\underset{b=1}{\sum }}tol\left({\hat{\beta }}_{1d\left(b\right)}\right)\right)}^{k}$ where tol is the tolerance associated to the parameter ${\hat{\beta }}_{1d\left(b\right)}$ , j refers to the direction (d=p for positive direction, d=n for negative direction) and k is chosen depending on the variability of the tolerance values: higher k is applied with lower variability to better discriminate between those models where there is higher collinearity between the positive and the negative index and those where collinearity is less severe. For example, we start with k=2 ; if the VIF for either WQS parameter exceeds 5 then we may increase k to, say, 3. In the following simulation and case studies we set k=3 since it allowed to find two indices with a VIF below 5.

To define the shrinkage parameter, lambda, a cross-validation step should be performed. However, since this can be computationally intense, a rule of thumb that can be applied to choose the value of the parameter lambda is to set it equal or close to the magnitude of the Akaike Information Criterion (AIC) of the non-penalized WQS regression. An outline of the steps to take when trying to fit a WQS regression with a single or double index follows:

- Step 1: fit a standard WQS regression (with single or double index as needed)
- Step 2: set three different shrinkage parameter values, one equal to the magnitude of the AIC of the regression fitted at step 1 one to a lower and one to a greater order of magnitude and follow a rough bisection algorithm. Alternatively, the search of the best lambda can also be refined by looking to the intermediate values, e.g., if the magnitude of the AIC is 1,000, then lambda can be set equal to 500 and 5,000 besides 100 and 10,000.
- Step 3: In the case of the 2iWQS a final check of the correlation between the two indices is needed. The exponent in the signal function can be increased in the case of multicollinearity.

The possibility to fit a 2iWQS and to estimate penalized weights will be added to the gWQS R package (23).

### Simulation study

**Figure 1:**
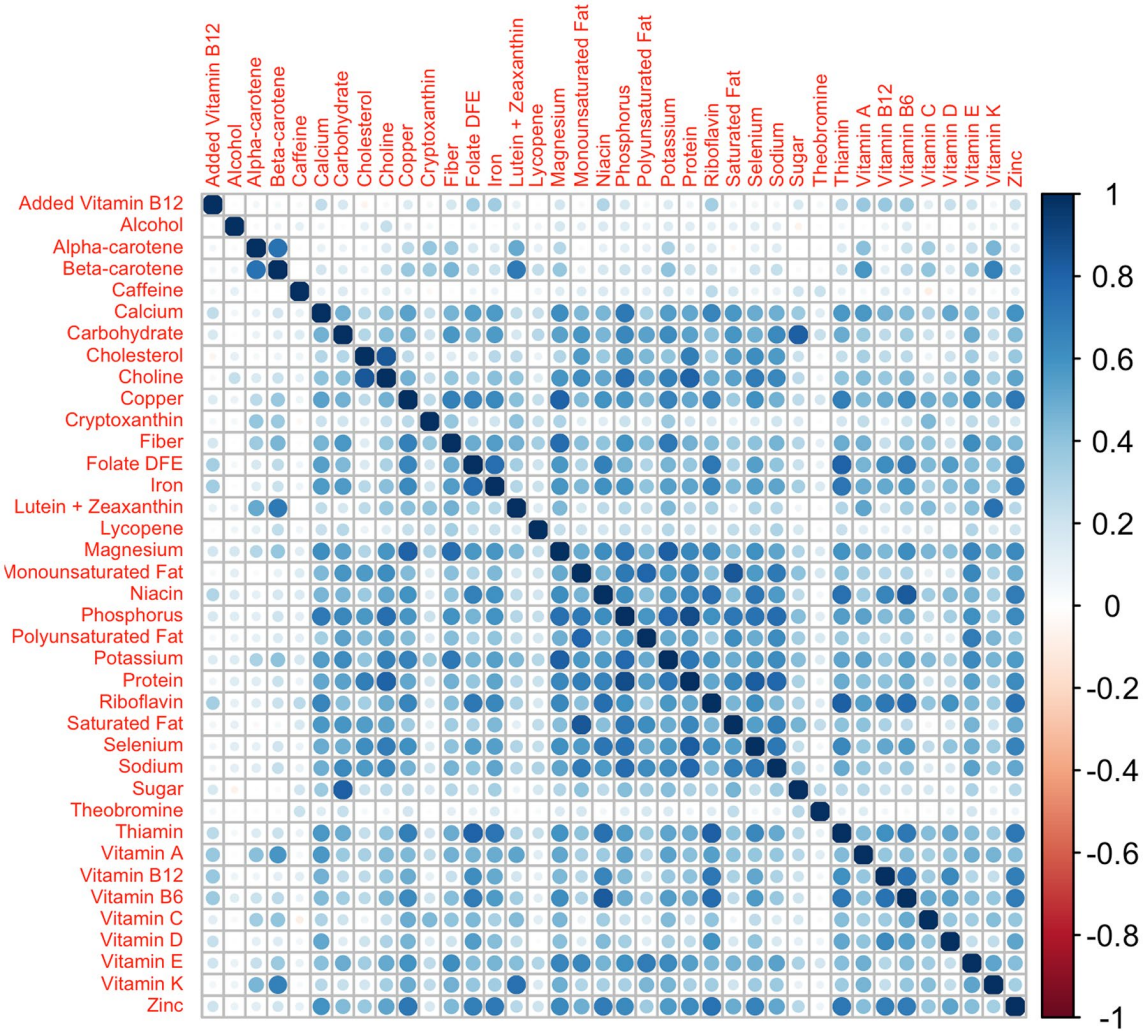
Nutrients’ correlation matrix: correlation matrix among the 38 nutrients from the NHANES 2011–2012, 2013–2014, and 2015–2016 survey cycles.

To evaluate the performance of this new model in terms of the estimation of the regression parameters and the weights, we performed a simulation study where we compared the results obtained by the 2iWQS with those obtained by the standard WQS regression and the quantile g-computation, a novel statistical method that combines WQS regression and g-computation to provide an effect estimate per simultaneous quantile increase in all elements (i.e., overall mixture effect), as well as weights that represent the importance of individual components of the mixture in the positive or negative direction (24). For a direct comparison with the directional sum mixture effect estimates ($\beta_{1d}$ ) of WQS regression, we will evaluate the analogous directional sum mixture effect estimates of the quantile g-computation models ($\psi_{d}$ ) rather than the overall mixture effect (ψ ). The former are calculated in quantile g-computation models as the sum of all mixture component coefficients in a given direction, while the latter is the sum of all mixture component coefficients (24). In all simulation scenarios, we have applied a repeated holdout approach (25) for all standard WQS regression and 2iWQS regression models to have more power in the parameter estimates and to reduce variability in the weights (26). We set 50 different training and validation splits of the dataset with 50 bootstrap iterations performed on each training set; we used 50 instead of the typical 100 or more to limit computational time. We took the data from the NHANES 2011–2012, 2013–2014, and 2015–2016 survey cycles where a total of 38 nutrients were measured through the administration of a food frequency questionnaire. In total 100 different datasets were built generating the 38 variables from a multivariate normal distribution keeping the same correlation structure of the original data. As we can see from Figure 1 the nutrient data show a complex correlation matrix with Spearman estimates ranging from −0.08 to 0.883.

Based on the case study results (see results section) the corresponding five nutrients with a median weight exceeding the threshold were selected for the positive direction while the corresponding 10 elements were chosen in the negative direction. In particular, the dependent variable was generated from a normal distribution with mean equal to the combination obtained by applying the parameters given in Table 1 to the 2iWQS formula and a unit standard deviation. The case study weights were rescaled assigning a null weight to all the non-selected nutrients. One more scenario was considered halving the values of the correlation matrix. This simulation study is structured such that the sensitivity in the positive direction (i.e., estimating weights exceeding the threshold of 1/38 = 0.026) is particularly difficult when two of the four nutrients have “true” weights of 0.05 and 0.07. As a last scenario, we considered a unidirectional association between the mixture and the outcome and we used only the positive weights to generate the dependent variable as described above.

**Table 1:** Parameters’ value used in the simulation study: values of the parameter regression and weights used to generate the dependent variable.

Parameter	PWQS	NWQS
β_1p_	0.5	
β_1n_		−0.5
$w_{sodium}$	0.50	0
$w_{polyunsaturated\;fat}$	0.19	0
$w_{cholesterol}$	0.15	0
$w_{caffeine}$	0.10	0
$w_{calcium}$	0.07	0
$w_{magnesium}$	0	0.27
$w_{fiber}$	0	0.13
$w_{vitamin\;C}$	0	0.12
$w_{vitamin\;B6}$	0	0.09
$w_{beta-carotene}$	0	0.08
$w_{folate\;DFE}$	0	0.08
$w_{vitamin\;D}$	0	0.07
$w_{vitamin\;E}$	0	0.06
$w_{vitamin\;K}$	0	0.06
$w_{alpha-carotene}$	0	0.05

The weight of all the remaining variables not included in the table were set to 0.

DFE, Dietary Folate Equivalents; PWQS, Positive Weighted Quantile Sum index; NWQS, Negative Weighted Quantile Sum index.

### Case study

We applied the new method of the 2iWQS regression to assess the effects of nutrients on obesity among adults using the NHANES 2011–2016 data. Consent from subjects participating in the study was received prior to conducting the study and the study has been reviewed and approved by the CDC/NCHS Ethics Review Board (ERB).

**Figure 2:**
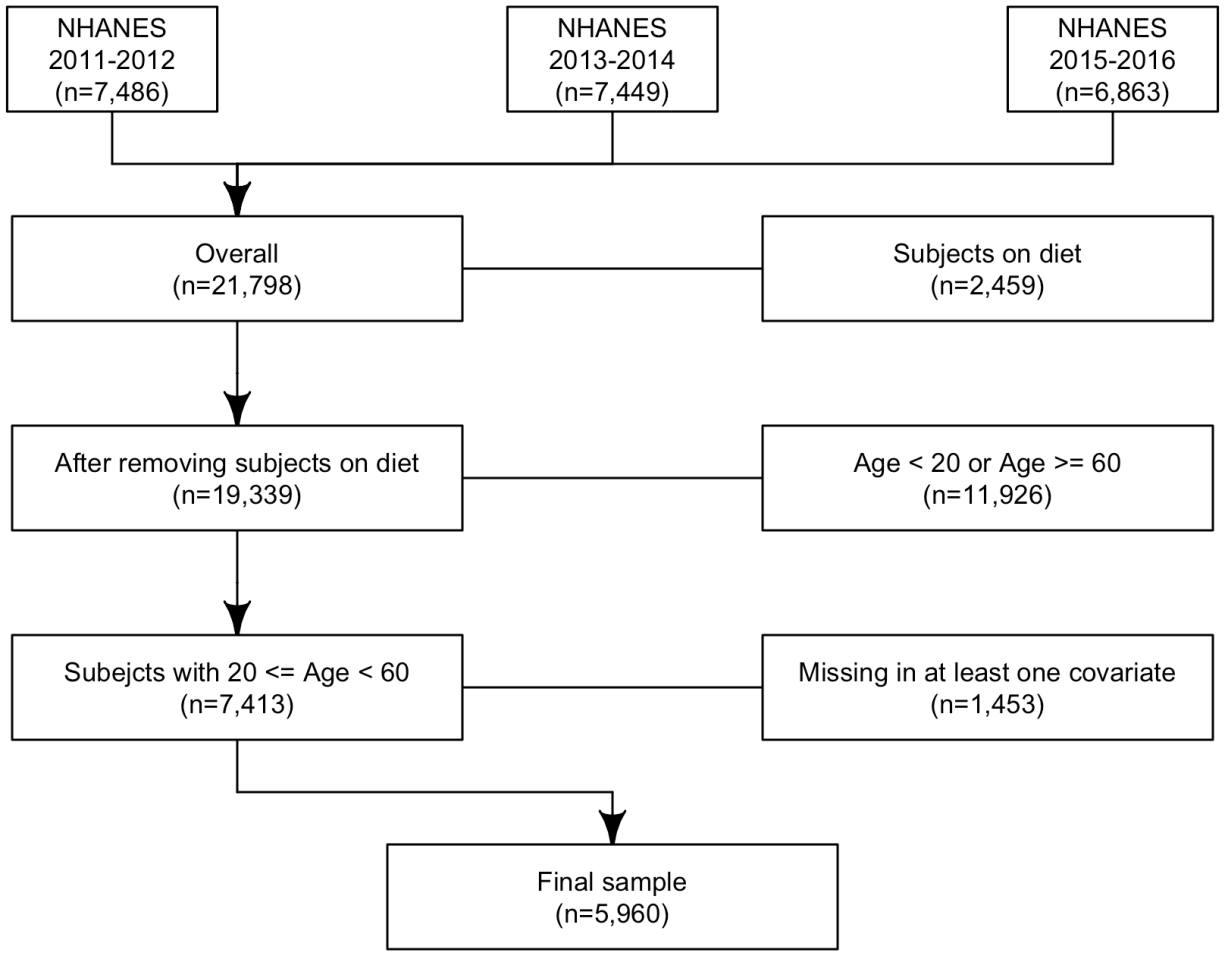
Analytic sample selection’s flowchart: flowchart of analytic sample selection in NHANES 2011–2016.

In total 21,798 subjects with reliable dietary data (meaning that all relevant variables associated with the 24-h dietary recall contain a value) were included in the analysis; 2,459 subjects were excluded because they were on any kind of diet to lose weight or for another health-related reason at the time of the interview; 11,926 subjects were younger than 20 or older than 60 years old and 1,453 subjects had a missing value at least for one of the covariates considered in the analysis. In total, 5,960 subjects were included in the study (Figure 2).

Obesity was defined as Body Mass Index (BMI) greater or equal to 30 kg/m^2^.

Nutrients were estimated from the dietary intake data that considered the types and amounts of foods and beverages (including all types of water) consumed during the 24-h period prior to the interview (midnight to midnight). Two interviews were performed: the first one was collected in-person while the second interview was collected by telephone 3–10 days later. Details of the survey are described elsewhere (27, 28). In this study, we averaged the two nutrients when both evaluations were considered as usual food consumption compared to the food habits of each participant (only one measurement was included in the analysis if the other one was not usual while the observation was dropped if both evaluations were not usual; this was a self-reported answer to the question of whether the person’s overall intake on the previous day was much more than usual, usual, or much less than usual) and we added the dietary supplement intake when applicable.

The models were adjusted by covariates including age, sex, race, education as the highest grade or level of school completed or the highest degree received (defined as a continuous variable score ranging from 1 = Less than 9th grade, to 5 = College graduate or above), the ratio of family income to poverty (using the Department of Health and Human Services poverty guidelines), the minutes of sedentary activity represented by the time spent sitting on a typical day and the minutes of moderate (defined as activity that causes small increases in breathing or heart rate and is done for at least 10 min continuously) and vigorous activities (defined as activity that causes large increases in breathing or heart rate for at least 10 min continuously) spent either during work or during recreational activities on a typical day categorized using its tertiles (because of the skewed distribution), the smoking status as never-smokers (subjects who did not smoke as many as 100 cigarettes in their lifetime), former smokers (those who smoked at least 100 cigarettes in their lifetime but were not currently smoking cigarettes), and current smokers (subjects that currently smoked cigarettes) and the study cycle.

The Kruskal-Wallis test and Chi-squared test were used to test differences between obese and non-obese participants for continuous and categorical variables, respectively. A 2iWQS regression with repeated holdout was fit to assess the effect of the nutrients on obesity. We set 100 different training and validation splits of the dataset and 100 bootstrap iterations performed on each training set. All simulation and case study analyses can be replicated through the code in the Supplementary material.

## Results

### Simulation study

**Figure 3:**
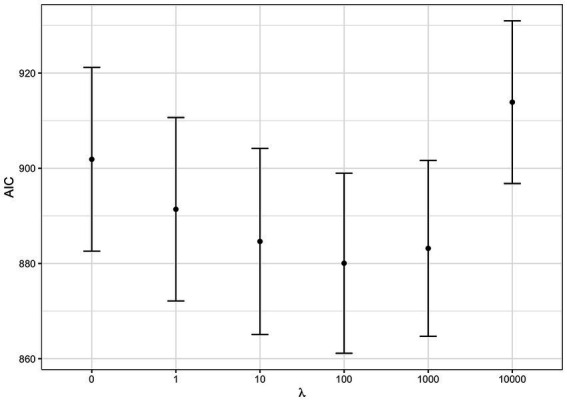
AIC at varying shrinkage parameters: WQS regression Akaike Information Criterion (AIC) at varying shrinkage parameter λ . The dots represent the average AIC obtained by fitting WQS models on the 100 datasets fixing λ to the corresponding value. Error bars are estimated as average AIC ± one standard error.

As a first step we tested for the best shrinkage parameter λ : a WQS regression was fitted on each dataset letting λ vary among the values 0, 1, 10, …, 10^4^. The best shrinkage parameter was selected which minimized the AIC. In our case, λ=100 was the optimum (i.e., smallest) value as shown in Figure 3.

**Figure 4:**
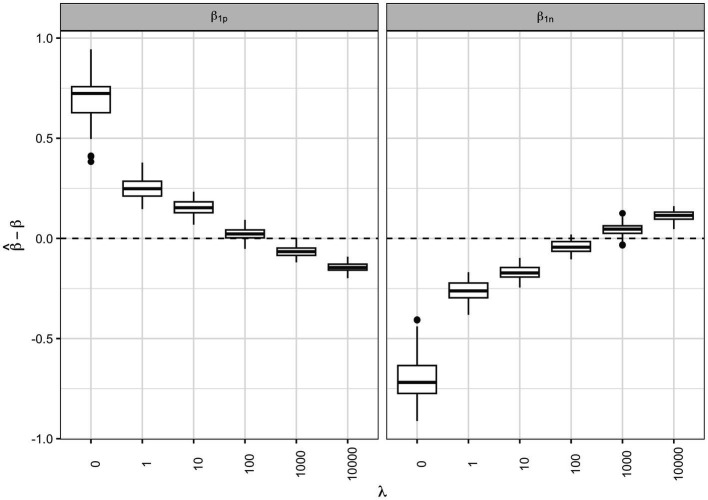
Regression parameter estimates at varying shrinkage parameters: box-plots of the bias associated to the $\beta_{1p}$ and $\beta_{1n}$ regression parameter estimates for the positive and negative direction, respectively, at different shrinkage parameters λ .

We then checked the accuracy of the parameter estimates at varying shrinkage values. In Figure 4 the bias of the regression parameters $\beta_{1p}$ and $\beta_{1n}$ is presented for the positive and the negative direction, respectively, at varying λ . The most accurate estimates for the regression parameters correspond to the shrinkage parameter that minimized the AIC (λ=100 ).

**Figure 5:**
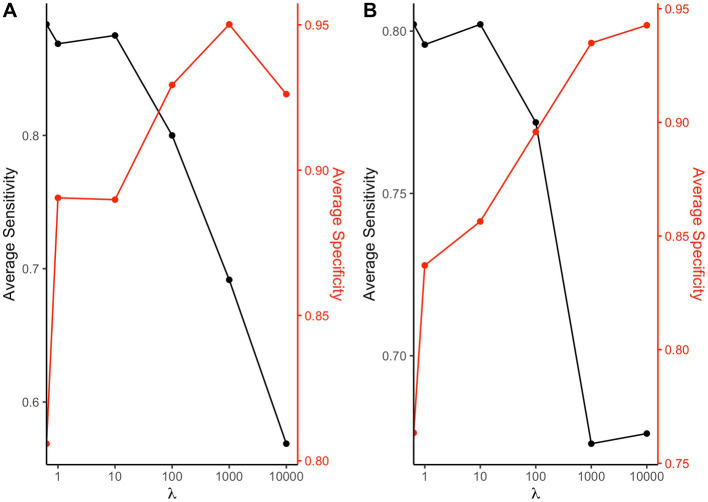
Sensitivity and specificity at varying shrinkage parameter: average sensitivity and specificity of the 2iWQS method in detecting the elements with a weight greater than 0 and those with null weight associated to a positive **(A)** or a negative **(B)** direction at varying λ .

We performed the same analysis for the weights: in Figure 5 we can see that when λ=100 we have accurate estimates both in terms of average sensitivity and specificity in identifying the elements truly associated with the outcome and those that do not have any relationship both for the positive (panel A) and negative (panel B) direction (positive direction: average sensitivity = 80.0%, average specificity = 92.9%; negative direction: average sensitivity = 77.2%, average specificity = 89.6%).

Once the optimum shrinkage parameter λ was identified, three additional regressions were fitted on each of the 100 datasets: in method 1 two separate WQS regressions without penalization were performed, one exploring the positive direction (i.e., where $\beta_{1}$ was constrained to be positive in the nonlinear estimation) and the second exploring the negative direction; in method 2 the WQS set of weights was estimated separately and without penalization for the positive and negative directions and once the WQS indices were estimated they were included in the same regression model fitted on the validation set; while in method 3 we applied quantile g-computation. We then compared these results with the ones obtained by applying the 2iWQS regression and penalized weights which we refer to as method 4.

**Figure 6:**
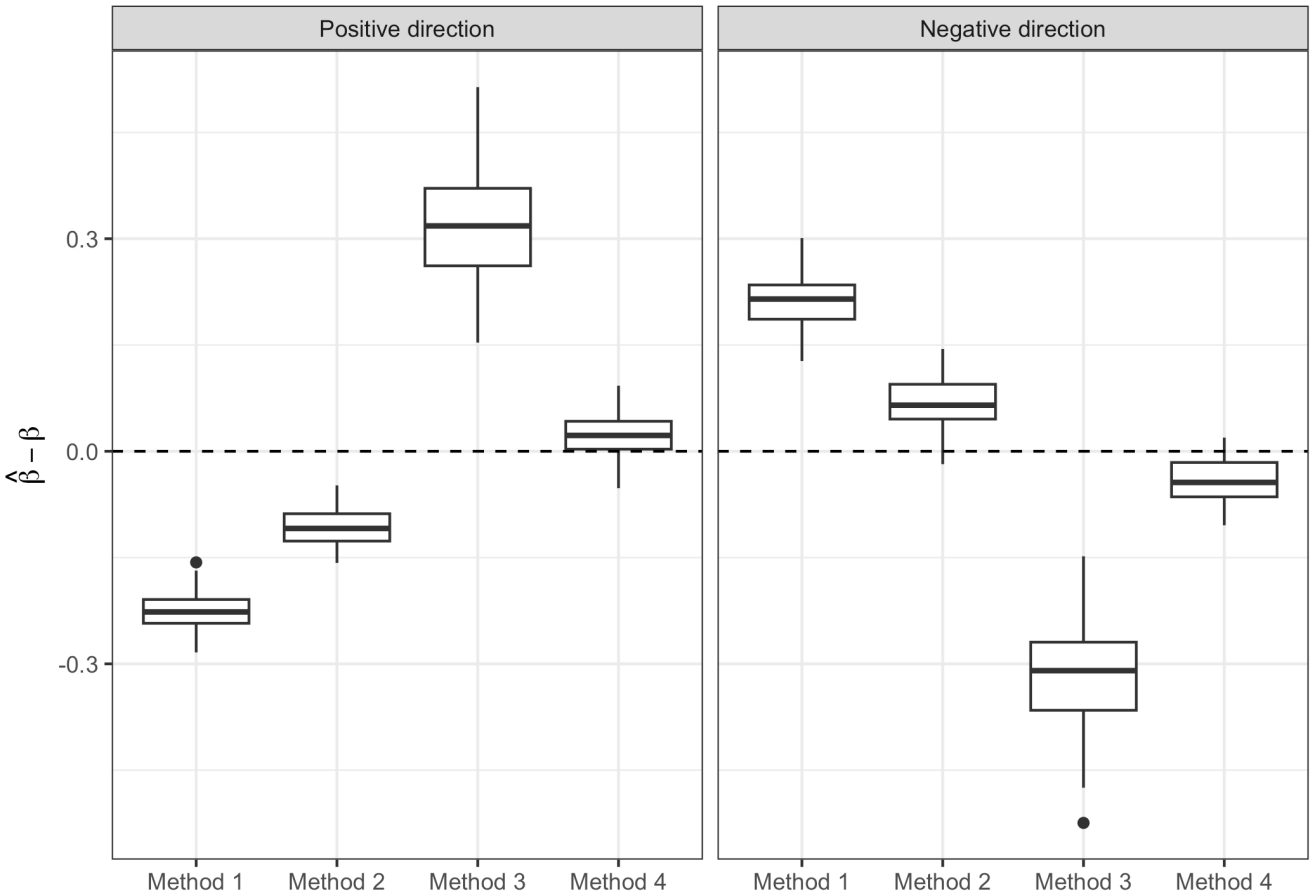
Regression parameter estimates of the three methods: box-plots of the bias in the regression parameter estimates associated with the two WQS indices of method 1, 2, and 4 and to the positive and negative ψ of quantile g-computation (method 3).

Figure 6 shows the box-plots of the bias associated with the $\beta_{1p}$ and $\beta_{1n}$ for method 1, 2 and 4 and $\psi_{1p}$ and $\psi_{1n}$ for method 3. We can see how method 4 is able to give a better estimate of the parameters [Positive direction Median Error (PME): 0.022, Standard error (SE): 0.029; Negative direction Median Error (NME): −0.044, SE: 0.031] compared to method 1 (PME: −0.227, SE: 0.028; NME: 0.215, SE: 0.037), method 2 (PME: −0.109, SE: 0.025; NME: 0.065, SE: 0.037) and method 3 (PME: 0.318, SE: 0.080; NME: 0.310, SE: 0.080).

To measure the performance of the 3 methods in identifying the elements associated with the outcome and those that do not have any relationship (in this case method 1 and 2 share the same weight estimates) we considered the average sensitivity and specificity. When we set a threshold equal to the inverse of the number of elements in the mixture to discriminate between the significant and non-significant weights, we observed that method 4 shows better sensitivity in both directions compared to methods 1 and 2 (positive direction: method 1–2 = 76.0%, method 4 = 80.0%; negative direction: method 1–2 = 59.0%, method 4 = 77.2%) while the specificity was similar in both directions (positive direction: method 1–2 = 92.5%, method 4 = 92.9%; negative direction: method 1–2 = 90.2%, method 4 = 89.6%) (Supplementary Figures S1, S2). Method 3 was not considered in these comparisons since in quantile g-computation a rule to identify the significant elements in the association is not defined.

The same analysis was performed in a different scenario where the correlation values among the elements in the mixture were halved. Similar results were obtained in this scenario compared to the one considering the original correlation matrix: best estimates were observed in estimating beta regression parameters in the new scenario for method 4 (PME: −0.018, SE: 0.042; NME: 0.028, SE: 0.052) and method 2 (PME: 0.001, SE: 0.035; NME: −0.037, SE:0.038), while method 1 (PME: −0.154, SE: 0.031; NME: 0.215, SE: 0.045) and method 3 (PME: 0.182, SE: 0.044; NME: −0.175, SE: 0.043) showed higher bias (Supplementary Figure S3).

When we looked at the ability of the methods in detecting the true elements with a non-null weight and those not associated with the outcome we observed a similar average sensitivity of method 4 and method 1–2 in both directions (positive direction: method 1–2 = 79.8%, method 4 = 80.2%; negative direction: method 1–2 = 76.5%, method 4 = 78.7%) but better specificity of method 4 compared to method 1–2 in both directions (positive direction: method 1–2 = 94.3%, method 4 = 97.0%; negative direction: method 1–2 = 88.8%, method 4 = 95.5%) (Supplementary Figures S4, S5).

In the last analysis, we tested the performance of the penalized weights when considering a unidirectional association between the mixture and the outcome. In this case, we applied an additional method that included a single index exploring the positive direction (method 4 1d) and a penalization term was fixed at λ=1000 . We note that in the 2iWQS regression model if no set of weights is found in one of the two directions, we let the function automatically compute the single index WQS regression. A warning message saying that no weight estimates are found in one of the two directions is shown by the function. Method 4 showed better estimates in both directions of the regression parameter associated with the WQS index (method 4, PME: −0.033, SE: 0.024; NME: −0.026, SE: 0.026) compared to method 1 (PME: 0.052, SE: 0.026; NME: 0.151, SE: 0.062), method 2 (PME: 0.097, SE: 0.031; NME: −0.094, SE: 0.023) and method 3 (PME: 0.420, SE: 0.083; NME: −0.414, SE: 0.078) (Supplementary Figure S6). To evaluate the weight estimates we only considered the positive direction: Method 4 showed lower sensitivity (method 1–2 = 84.6%, method 4 = 60.8%) (Supplementary Figure S7) but similar specificity compared to methods 1–2 (method 1–2 = 92.1%, method 4 = 92.7%) (Supplementary Figure S8). Since no association was found in the negative direction (only in 1% of the scenarios we observed a false positive through method 4) we applied method 4 considering a single index in the positive direction (method 4 1d): a better estimate of the regression parameter was found (ME: −0.009, SE: 0.021) (Supplementary Figure S6) as well as a higher sensitivity (78.5%) (Supplementary Figure S7) and specificity (96.1%) (Supplementary Figure S8) compared to method 4, suggesting that if the association between the mixture and the dependent variable was not significant in one of the two directions, then a model with single index (the one exploring the significant direction) should be fitted as a final model to have more accurate estimates.

### Case study

In total 5,960 subjects were included in the case study analysis. Table 2 shows the descriptive statistics of the overall population and divided by obese and non-obese for the covariates included in the 2iWQS regression. A total of 2,158 (36.2%) subjects were obese and were characterized by a higher median age and higher prevalence of females compared to non-obese participants. A different race distribution was also observed showing a higher percentage of Mexican and Black subjects and a lower prevalence of Asian participants among obese. Finally, a lower level of education was detected among obese people as well as a lower income to poverty ratio index, a higher number of minutes spent in sedentary activities and a higher frequency of low time spent performing moderate to vigorous-intensity activities. Summary statistics related to the 38 nutrients included in the analysis are shown in Table 3. All the elements that showed a significant difference between the two groups had higher values among non-obese subjects apart from caffeine.

**Table 2:** Sociodemographics and lifestyles: descriptive statistics of the variables included in the study for the overall population and divided by obese and non-obese.

	Non-obese (*N* = 3,802)	Obese (*N* = 2,158)	Total (*N* = 5,960)	*p* value
Median (Q1, Q3)				
Age	38.0 (28.0, 48.0)	41.0 (32.0, 50.0)	39.0 (30.0, 49.0)	**<0.001**
Education	4.0 (3.0, 5.0)	4.0 (3.0, 4.0)	4.0 (3.0, 5.0)	**<0.001**
Family income to poverty ratio	2.4 (1.1, 4.6)	1.9 (1.0, 3.7)	2.2 (1.1, 4.2)	**<0.001**
Minutes of sedentary activity	360.0 (240.0, 480.0)	360.0 (240.0, 540.0)	360.0 (240.0, 480.0)	**<0.001**
Count (%)				
Sex				**<0.001**
Males	2003 (52.7%)	961 (44.5%)	2,964 (49.7%)	
Females	1799 (47.3%)	1,197 (55.5%)	2,996 (50.3%)	
Race				**<0.001**
Asian	703 (18.5%)	84 (3.9%)	787 (13.2%)	
Black	671 (17.6%)	564 (26.1%)	1,235 (20.7%)	
Mexican	428 (11.3%)	374 (17.3%)	802 (13.5%)	
Other Hispanic	351 (9.2%)	209 (9.7%)	560 (9.4%)	
Others	140 (3.7%)	87 (4.0%)	227 (3.8%)	
White	1,509 (39.7%)	840 (38.9%)	2,349 (39.4%)	
Moderate and vigorous-intensity activities				**<0.001**
Low	1,251 (32.9%)	887 (41.1%)	2,138 (35.9%)	
Medium	1,312 (34.5%)	592 (27.4%)	1,904 (31.9%)	
High	1,239 (32.6%)	679 (31.5%)	1,918 (32.2%)	
Smoking status				0.399
Never	2,320 (61.0%)	1,283 (59.5%)	3,603 (60.5%)	
Former	615 (16.2%)	375 (17.4%)	990 (16.6%)	
Current	867 (22.8%)	500 (23.2%)	1,367 (22.9%)	

Median, 1st (Q1) and 3rd quartiles (Q3) are shown for continuous variables while counts and percentages were considered for categorical variables. Kruskal-Wallis test and Chi-squared test were used to test for differences for continuous and categorical variables, respectively. Bolded *p*-values highlight the statistically significant results.

**Table 3:** Nutrient characteristics: summary statistics of the 38 nutrients included in the analysis.

	Non-obese (*N* = 3,802)	Obese (*N* = 2,158)	Total (*N* = 5,960)	*p* value
Added vitamin B12 (mcg)	0.0 (0.0, 1.2)	0.0 (0.0, 0.9)	0.0 (0.0, 1.1)	**0.005**
Alcohol (gm)	0.0 (0.0, 7.8)	0.0 (0.0, 0.0)	0.0 (0.0, 5.6)	**<0.001**
Alpha-carotene (mcg)	70.0 (20.0, 443.4)	51.0 (15.0, 248.0)	62.2 (18.0, 370.2)	**<0.001**
Beta-carotene (mcg)	1091.5 (417.6, 2998.8)	798.0 (338.9, 2041.6)	975.0 (382.0, 2565.0)	**<0.001**
Caffeine (mg)	94.0 (25.5, 190.0)	100.2 (29.0, 204.4)	96.0 (27.5, 193.0)	**0.022**
Calcium (mg)	958.8 (656.1, 1358.4)	941.0 (639.6, 1316.6)	952.1 (650.9, 1347.5)	0.120
Carbohydrate (gm)	254.7 (190.2, 332.9)	245.0 (183.9, 318.8)	250.2 (188.3, 327.3)	**0.001**
Cholesterol (mg)	254.5 (158.0, 393.9)	262.0 (164.0, 410.0)	256.5 (160.0, 400.0)	0.050
Total choline (mg)	314.1 (224.0, 434.8)	302.4 (213.2, 423.4)	310.6 (220.0, 430.5)	**0.002**
Copper (mg)	1.3 (0.9, 1.8)	1.1 (0.8, 1.6)	1.2 (0.9, 1.8)	**<0.001**
Beta-cryptoxanthin (mcg)	38.5 (12.5, 101.0)	37.0 (12.5, 93.5)	37.5 (12.5, 98.5)	0.258
Dietary fiber (gm)	16.4 (11.2, 23.9)	14.6 (10.1, 21.0)	15.8 (10.7, 22.8)	**<0.001**
Folate, DFE (mcg)	606.8 (403.5, 949.7)	527.0 (345.1, 829.1)	578.0 (381.0, 908.8)	**<0.001**
Iron (mg)	15.1 (10.9, 21.7)	14.0 (10.0, 20.5)	14.7 (10.5, 21.2)	**<0.001**
Lutein + zeaxanthin (mcg)	891.2 (474.5, 1824.0)	785.2 (424.6, 1471.2)	854.8 (455.0, 1681.2)	**<0.001**
Lycopene (mcg)	2536.5 (644.0, 6611.5)	2498.8 (600.0, 6545.4)	2525.5 (622.8, 6598.8)	0.564
Magnesium (mg)	307.0 (229.5, 414.0)	281.0 (202.8, 370.0)	297.4 (219.0, 397.6)	**<0.001**
Total monounsaturated fatty acids (gm)	26.9 (18.7, 36.5)	26.8 (19.2, 36.2)	26.9 (18.9, 36.4)	0.844
Niacin (mg)	28.5 (20.2, 40.4)	26.6 (18.8, 37.7)	27.8 (19.7, 39.4)	**<0.001**
Phosphorus (mg)	1352.2 (1022.6, 1763.0)	1295.5 (986.6, 1723.4)	1332.8 (1009.0, 1745.5)	**0.003**
Total polyunsaturated fatty acids (gm)	17.4 (11.9, 24.3)	17.6 (12.2, 24.8)	17.4 (12.0, 24.5)	0.375
Potassium (mg)	2598.2 (1954.2, 3360.9)	2399.5 (1818.2, 3086.4)	2526.2 (1898.6, 3281.9)	**<0.001**
Protein (gm)	81.7 (61.3, 107.5)	78.0 (58.3, 102.5)	80.5 (60.1, 105.7)	**<0.001**
Riboflavin (Vitamin B2) (mg)	2.2 (1.5, 3.3)	2.1 (1.4, 3.1)	2.1 (1.5, 3.3)	**<0.001**
Total saturated fatty acids (gm)	24.0 (16.2, 34.1)	24.4 (17.1, 34.3)	24.2 (16.4, 34.2)	0.081
Selenium (mcg)	122.8 (89.0, 166.4)	116.2 (84.2, 159.8)	120.6 (87.1, 163.9)	**<0.001**
Sodium (mg)	3477.2 (2582.1, 4578.4)	3401.2 (2585.0, 4410.8)	3456.8 (2582.9, 4522.5)	0.110
Total sugars (gm)	100.4 (65.6, 145.2)	102.9 (66.7, 148.1)	101.1 (66.0, 146.4)	0.277
Theobromine (mg)	7.5 (0.0, 41.5)	7.0 (0.0, 40.5)	7.5 (0.0, 41.0)	0.184
Thiamin (Vitamin B1) (mg)	1.8 (1.3, 2.7)	1.6 (1.1, 2.4)	1.7 (1.2, 2.6)	**<0.001**
Vitamin A, RAE (mcg)	517.0 (304.5, 816.0)	456.8 (275.1, 724.2)	495.0 (293.4, 774.0)	**<0.001**
Vitamin B12 (mcg)	5.5 (3.1, 10.6)	5.1 (2.9, 9.7)	5.3 (3.0, 10.3)	**0.003**
Vitamin B6 (mg)	2.4 (1.6, 3.9)	2.1 (1.4, 3.4)	2.3 (1.5, 3.7)	**<0.001**
Vitamin C (mg)	86.6 (37.5, 169.6)	70.5 (28.5, 143.1)	81.3 (33.9, 158.1)	**<0.001**
Vitamin D (D2 + D3) (mcg)	5.6 (2.3, 13.7)	4.6 (2.1, 11.2)	5.2 (2.2, 12.9)	**<0.001**
Vitamin E as alpha-tocopherol (mg)	7.7 (5.3, 11.3)	7.1 (4.9, 10.4)	7.5 (5.2, 10.9)	**<0.001**
Vitamin K (mcg)	87.0 (51.4, 154.0)	74.8 (44.5, 127.9)	82.7 (48.9, 143.3)	**<0.001**
Zinc (mg)	12.3 (8.4, 18.3)	11.6 (7.8, 17.1)	12.0 (8.2, 17.7)	**<0.001**

Median, 1st and 3rd quartiles are shown for the overall population and divided in obese and non-obese participants. Kruskal-Wallis test was applied to test for differences between the two groups. Bolded *p*-values highlight the statistically significant results.

**Figure 7:**
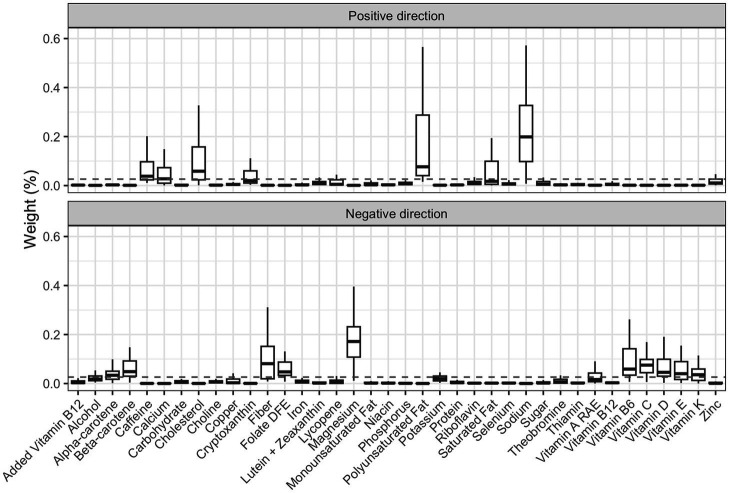
Box-plot of the weights associated to the positive and negative index estimate through the repeated holdout 2iWQS regression. The dashed line represents the prespecified cut-off established to identify the most important elements of the mixture and set equal to the inverse of the number of the mixture components (0.026).

We then applied the 2iWQS regression to test for the association between the nutrients and the outcome adjusting for all the covariates reported in Table 2. We used a repeated holdout approach to have more stable results including all the observations in the study to estimate both the weights and the regression parameters during the repeated testing and validation steps (25). A total of 100 repeated holdout 2iWQS regressions were performed. Table 4 shows the effects and their 95% Confidence Intervals (CIs) of both the positive and the negative index on the probability of being obese. Both indices were associated with the outcome. The median was used as the parameter point estimates while the 2.5th and the 97.5th percentiles were considered to build the 95% CIs. In Table 4 are also shown the medians of the weights greater than the prespecified cutoff (1/38 = 0.026) for the positive and negative index where sodium and magnesium showed a predominant role in the positive and negative association with obesity, respectively. However, there is also an indication of a mixture effect in the positive and in the negative direction including other components with medians above the cutoff threshold. The distributions of all the elements included in the analysis estimated for both indices are provided in Figure 7.

**Table 4:** 2iWQS regression results: the estimates and 95% confidence intervals (CI) of the positive (PWQS) and negative (NWQS) indices are shown. The second part of the table shows the magnitude of the weights greater than the prespecified cutoff (0.026) for both the positive and the negative index.

	Estimate	95% CI
PWQS	0.107	0.051; 0.166
NWQS	−0.156	−0.194; −0.101
	Weights	
PWQS		
Sodium	0.199	
Polyunsaturated fat	0.077	
Cholesterol	0.059	
Caffeine	0.038	
Calcium	0.028	
NWQS		
Magnesium	0.172	
Fiber	0.081	
Vitamin C	0.075	
Vitamin B6	0.059	
Beta-carotene	0.049	
Folate DFE	0.048	
Vitamin D	0.046	
Vitamin E	0.040	
Vitamin K	0.036	
Alpha-carotene	0.034	

The model was adjusted for age, sex, race, education, the ratio of family income to poverty, the minutes of sedentary activity, the minutes of moderate and vigorous activities and the smoking status. CI, Confidence Interval; DFE, Dietary Folate Equivalents; PWQS, Positive Weighted Quantile Sum index; NWQS, Negative Weighted Quantile Sum index.

## Discussion

Through this work we were able to extend WQS regression to the case where the mixture considered in the study can have both a positive and a negative effect, moreover, we increased the ability of WQS in detecting the association between the mixture and the outcome as well as in identifying the true elements through the introduction of penalized weight estimates. The new method estimates two indices in the same regression model both including all the elements of the mixture, one constrained to be positive and one to be negative. A recent work from Keil and others (24) introduced a new approach that estimates the overall effect of the mixture on the outcome when there is uncertainty about the effect direction of some exposures. Differently from the original WQS regression, they proposed to estimate positive and negative weights within the same index using normalized linear (or generalized linear) regression coefficients and then estimating the effect of the overall mixture via a standard g-computation algorithm. With the q g-comp approach, the overall effect of the mixture is estimated (which is not the mixture effect) on the outcome, but we cannot estimate the impact in the positive and negative directions, especially with highly correlated components. Through our method, we introduced the possibility to measure both the beneficial and harmful effects of the exposure to the mixture separately keeping the advantages of the identification of a weighted index that allows increasing the power to detect the mixture effect and avoiding incurring the reversal paradox. This ability of the 2iWQS is at the expense of a small bias in the estimate of the regression parameter; however, we were able to almost null the bias due to the inclusion of both indices in the same model and the penalized estimates of the weights. Another advantage that we carry from WQS regression compared to other shrinkage methods is the ability to estimate a mixture effect, in the 2iWQS case in both directions, and with highly correlated variables to avoid a grouping effect like in elastic net or the arbitrary selection of variables like in LASSO that can be problematic in the risk evaluation of environmental mixture (19).

In this work, we showed how the two indices were built and how we deal with the correlation between the two indices. This was the main issue that we encountered: because of the high correlation among the elements included in the mixture we noticed a risk of collinearity when including both indices in the same regression model. To address this problem, we applied two strategies. As a first solution, we introduced a penalization parameter in the objective function to estimate the weights allowing us to better discriminate between the elements that have a weight significantly different from zero and those that have a null weight. This reduces the noise produced by the elements that are not associated with the outcome which can increase the correlation between the two indices. The second solution to reduce the correlation between the two indices was the application of the weighted mean based on the tolerance of each bootstrapped model in the estimates of the final weights. This gave more importance to the set of weights that produces less correlated indices.

In the real case study, we applied this new methodology by taking data from the NHANES 2011–2012, 2013–2014, and 2015–2016 study cycles to test for the association between nutrients and obesity. The results showed a harmful effect of sodium, polyunsaturated fatty acids, cholesterol, caffeine and calcium. While some nutrients like sodium (29–31) and cholesterol (32, 33) are already known risk elements for obesity, we also found nutrients for which there is controversial evidence of their effect on obesity. Because of the unavailable information on the different types of polyunsaturated fatty acids we were not able to disentangle which component drove the harmful effect on obesity. Polyunsaturated fatty acids are known to be protective against overweight and obesity (34–39), however, there is evidence that an increased intake of omega-6 long-chain polyunsaturated fatty acids can increase the risk of obesity (35, 40) in particular if there is an unbalanced omega-6/omega-3 ratio, an increasingly widespread problem in western countries (41). Calcium intake was observed to be a protective factor against obesity (42–45) but few studies did not show any effect (46, 47) or a harmful relationship (48). Finally, caffeine has a protective effect on a regular intake (49) but in excessive doses, it can affect insomnia and anxiety (50, 51) which are associated in turn with obesity (52–54). On the other hand, a protective effect against obesity was found for magnesium, fiber, vitamin C, vitamin B6, beta-carotene, folate Dietary Folate Equivalents (DFE), vitamin D, vitamin E, vitamin K and alpha-carotene. For these nutrients there was evidence of a beneficial effect against obesity in previous studies (55–70).

One strength of this novel approach is the ability to include all nutrients in the analysis considering the possible confounding that can be caused by the exclusion of some elements while previous studies showed the association of one or few elements at a time with obesity. Moreover, we showed that considering two indices in the same WQS regression with the penalization term increased the accuracy of the parameter estimates when the mixture has a bidirectional effect on the outcome of interest. In addition to already available methods like the quantile-based g-computation approach, our new methodology allows us to measure the double association of the mixture quantifying the effect in both the positive and negative direction with the dependent variable. As for WQS regression, a further advantage of this approach is the ease of use and of interpretation of the results due to the building of the indices representing the mixture exposure and the weights attributed to each element identifying the contribution of each element to the estimated association with the outcome. One additional advantage to be considered is the possibility to integrate this new feature of WQS in the random subset (21) and repeated holdout (25) extensions.

In contrast to the simplicity of the method, we can identify as a limitation of the WQS regression the lower flexibility due to the assumption of a linear trend between each element and the dependent variable. Before applying this method, it is recommended to check in advance if a non-linear trend exists between the mixture components and the outcome by adding a quadratic term in the WQS model to test for curvilinearity or by applying other regression methods that can deal with environmental mixtures like Bayesian Kernel Machine Regression (71).

## Conclusion

Through this work, we introduced an extension of WQS regression that improved the accuracy of the parameter estimates in WQS with a single index as well as when considering a mixture of elements where a subset may have a protective effect and other components may have a harmful effect on the outcome. This was possible through the application of penalized weight estimates and the introduction of a second index, allowing one WQS index to investigate the positive and one the negative direction of the association with the dependent variable. The usage of two indices in the same model quantifies the effect of the mixture in the two directions keeping them separate. This can be of interest in fields like nutrition where a measure of the protective and harmful effects of nutrients can help in dietary decision making.

## Data availability statement

Publicly available datasets were analyzed in this study. This data can be found at: https://cran.r-project.org/web/packages/gWQS/index.html.

## Ethics statement

The studies involving human participants were reviewed and approved by NCHS Research Ethics Review Board. The patients/participants provided their written informed consent to participate in this study.

## Author contributions

SR gave substantial contributions to the conception, the analysis and interpretation of data for the work, drafted the work and revised it critically for important intellectual content, and provided approval for publication of the content. CG and SC gave substantial contributions to the conception and interpretation of data for the work, revised the work critically for important intellectual content, and provided approval for publication of the content. All authors contributed to the article and approved the submitted version.

## Funding

SR was supported by Programma Operativo Nazionale “Ricerca e Innovazione” 2014–2020 (PON R&I FSE-REACT EU), Azione IV.4 “Contratti di ricerca su tematiche dell’innovazione.” CG was supported by the National Institute of Environmental Health Sciences (#U2CES026555, #P30ES023515, and #R01ES028811). SC was supported by research grants from the Italian Ministry of University (PRIN projects n. 20178S4EK9).

## Conflict of interest

The authors declare that the research was conducted in the absence of any commercial or financial relationships that could be construed as a potential conflict of interest.

## Publisher’s note

All claims expressed in this article are solely those of the authors and do not necessarily represent those of their affiliated organizations, or those of the publisher, the editors and the reviewers. Any product that may be evaluated in this article, or claim that may be made by its manufacturer, is not guaranteed or endorsed by the publisher.
