# Erratum: A weighted quantile sum regression with penalized weights and two indices

**DOI:** 10.3389/fpubh.2023.1289579

**Published:** 2023-09-28

**Authors:** Frontiers Production Office

**Affiliations:** Frontiers Media SA, Lausanne, Switzerland

**Keywords:** environmental mixture, weighted quantile sum regression, two indices, penalized weights, nutrients, obesity

Due to a production error, there was a mistake in the figure and table ordering as published in the HTML and XML files. The correct order appears in the final PDF file. Corrections were only required in the HTML and XML files. The publisher apologizes for the mistake. The original article has been updated.

