# Supplementary material for: A weighted quantile sum regression with penalized weights and two indices
Source: Front Public Health. 2023 Jul 18;11:1151821. doi: 10.3389/fpubh.2023.1151821 (PMC10392701; doi:10.3389/fpubh.2023.1151821)

Supplementary Material

A weighted quantile sum regression with penalized weights and two indices

Stefano Renzetti^*^, Chris Gennings, Stefano Calza

*** Correspondence:** Stefano Renzetti^*^ [stefano.renzetti@unibs.it](mailto:stefano.renzetti@unibs.it)

**Figure S1**: Heatmap of the sensitivity of the three methods in detecting the elements with a weight greater than 0 associated to a positive (panel A) or a negative (panel B) direction.


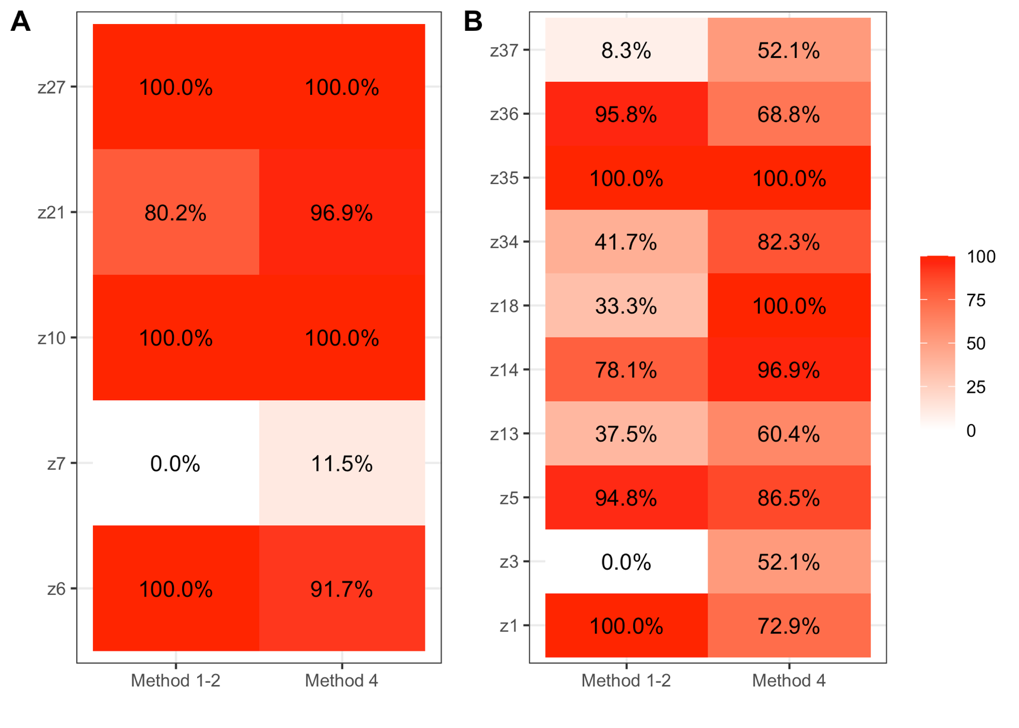


**Figure S2**: Heatmap of the specificity of the three methods in detecting the elements with a null weight associated to a positive (panel A) or a negative (panel B) direction.

**
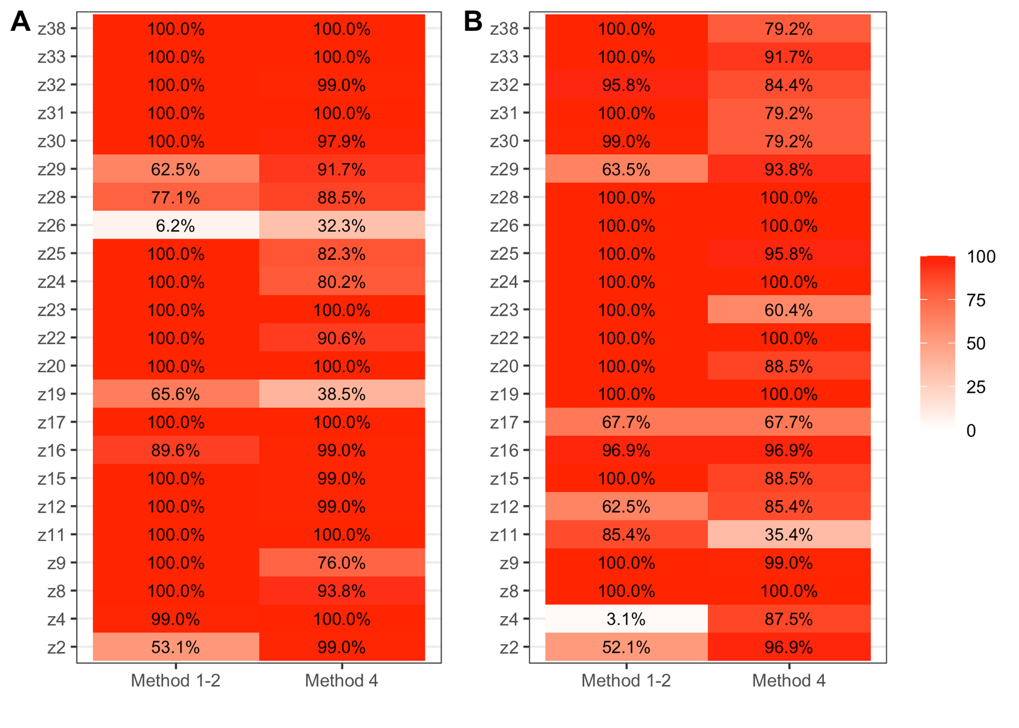
**

**Figure S3**: Box-plots of the bias in the estimates of the regression parameter associated to the two WQS indices of the four methods in scenario 2 where the correlation values were halved.


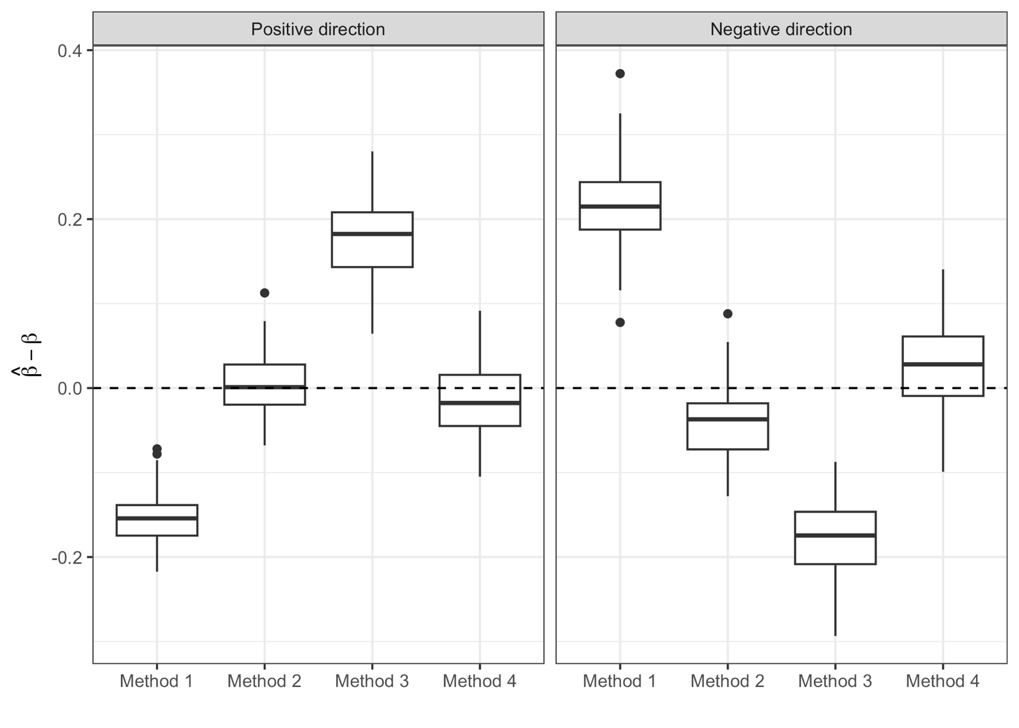


**Figure S4**: Heatmap of the sensitivity of the three methods in detecting the elements with a weight greater than 0 associated to a positive (panel A) or a negative (panel B) direction in scenario 2.


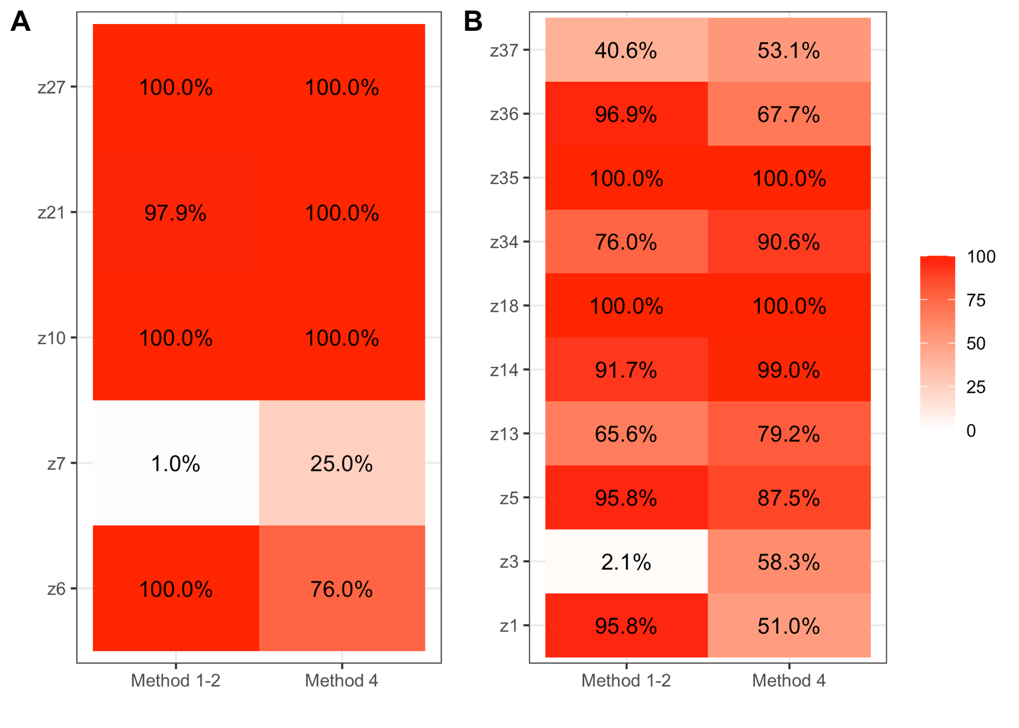


**Figure S5**: Heatmap of the specificity of the three methods in detecting the elements with a null weight associated to a positive (panel A) or a negative (panel B) direction in scenario 2.


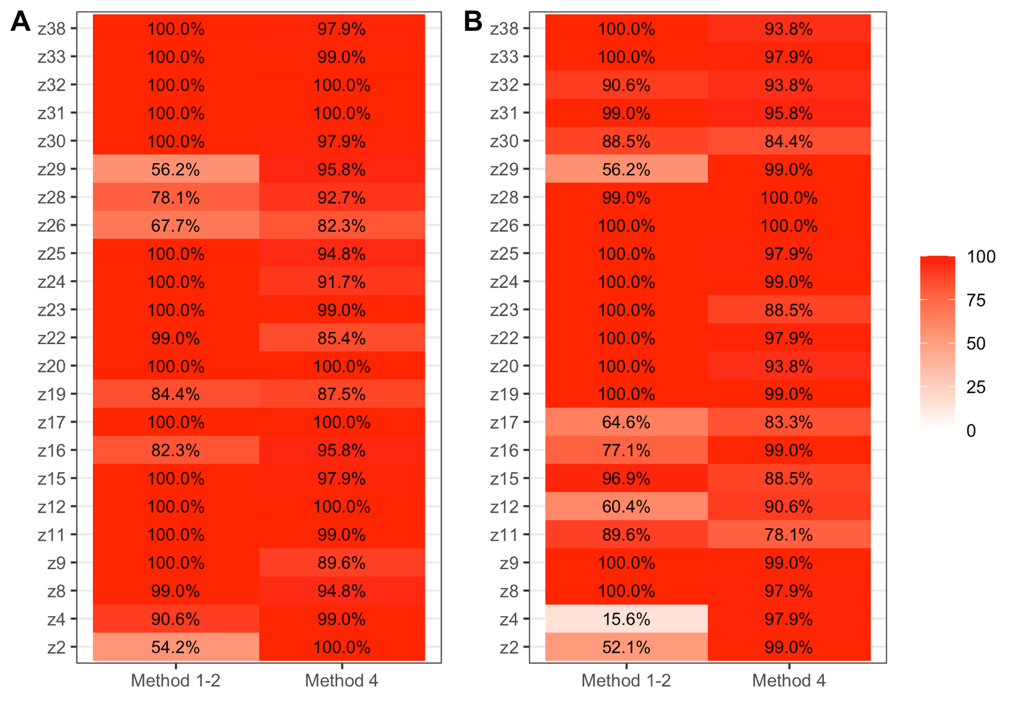


**Figure S6**: Box-plots of the bias in the estimates of the regression parameter associated to the two WQS indices of the four methods in scenario 3 where an association between the mixture and the outcome in a single (positive) direction was defined. The bias related to the estimates of the single positive index with penalized weights method is also displayed (method 4 1d).


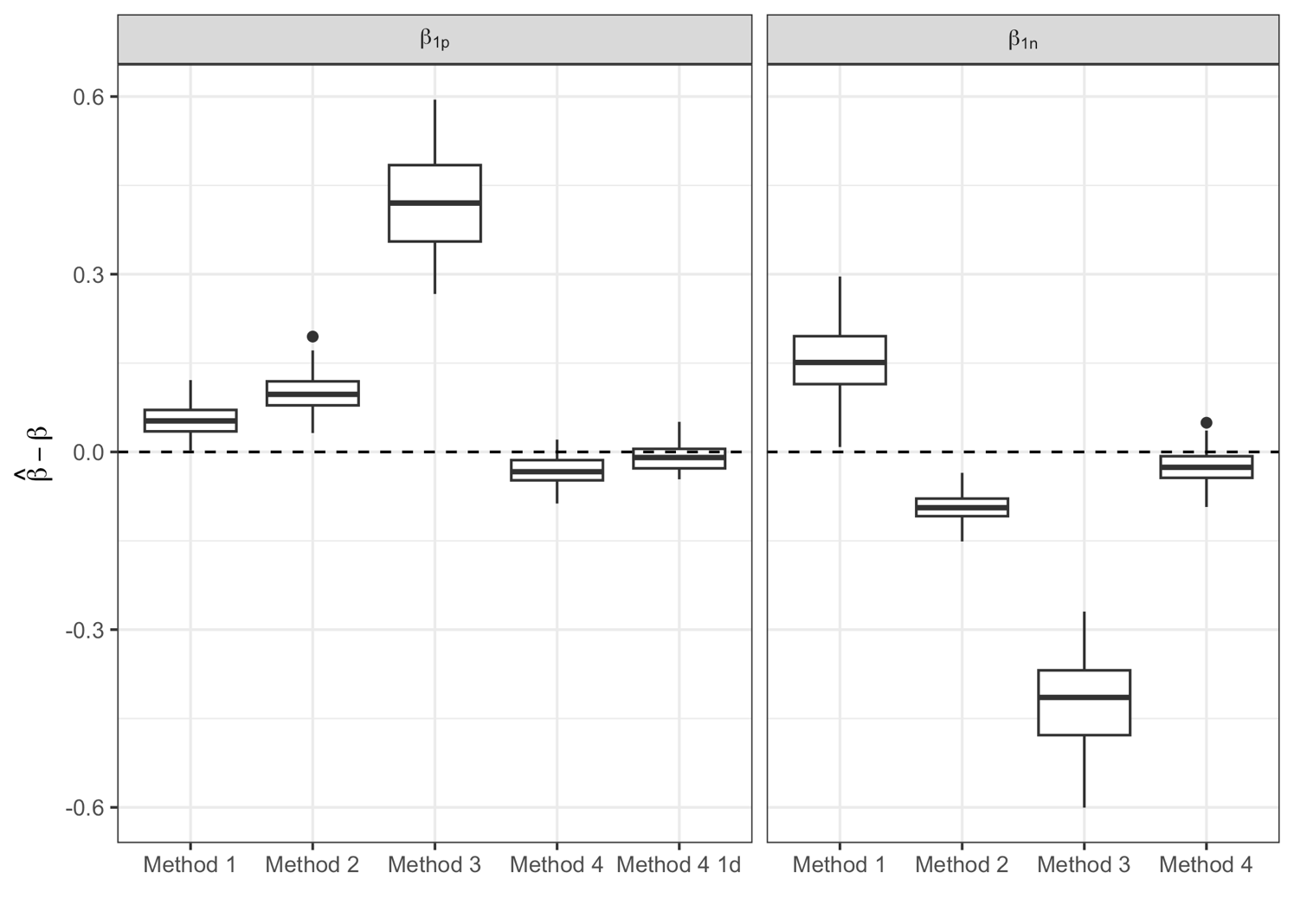


**Figure S7**: Heatmap of the sensitivity of method 1-2, method 4 and method 4 with single index (method 4 1d) in detecting the elements with a weight greater than 0 when there is a unidirectional effect of the mixture.


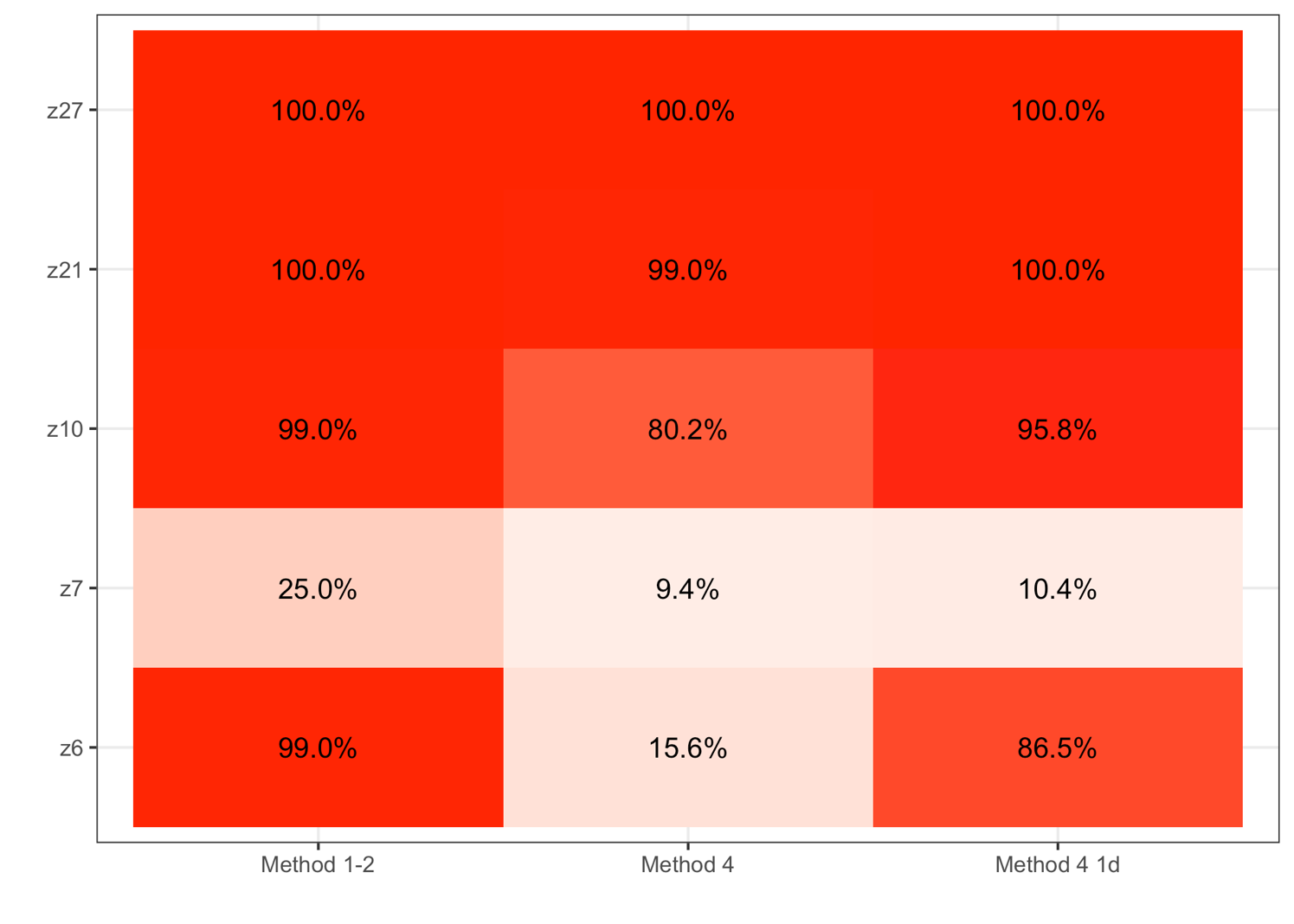


**Figure S8**: Heatmap of the specificity of method 1-2, method 4 and method 4 with single index (method 4 1d) in detecting the elements with a null weight when there is a unidirectional effect of the mixture.


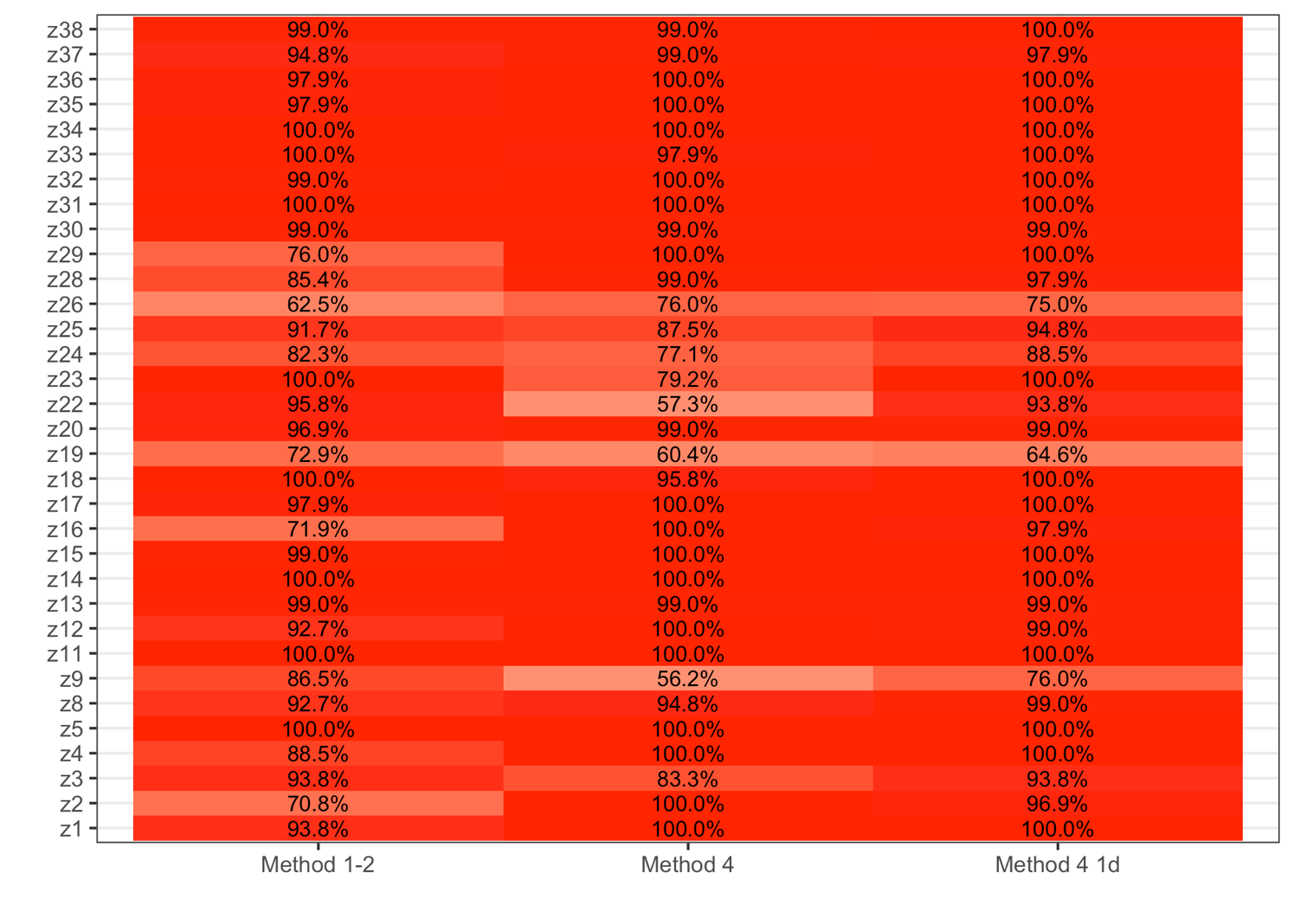

Supplement: Supplementary file 1 [file Table_1.docx]
