# Supplementary material for: A weighted quantile sum regression with penalized weights and two indices
Source: Front Public Health. 2023 Jul 18;11:1151821. doi: 10.3389/fpubh.2023.1151821 (PMC10392701; doi:10.3389/fpubh.2023.1151821)
Supplement: Supplementary file 3 [file Data_Sheet_2.docx]

***Replicable R code***

install.packages("pacman")

install.packages("devtools")

pacman::p_load(future, future.apply, dplyr, reshape2, MASS, qgcomp, gWQS)

# load the NHANES nutrition, outcome and covariates data

nutrients <- c("prot", "carb", "sugar", "fiber", "sfat", "mfat", "pfat", "chol", "alcol", "atoc",

"vara", "acar", "bcar", "cryp", "lyco", "lz", "vb1", "vb2", "niac", "vb6", "fdfe",

"chl", "vb12", "b12a", "vc", "vd", "vk", "calc", "phos", "magn", "iron", "zinc", "copp", "sodi", "pota",

"sele", "caff", "theo")

nutrients <- sort(nutrients)

##############

# Case study #

##############

# for computational time reasons we decreased the number of iterations

# In the paper the nuber of bootstraps and repeated holdout were set to b=100 and rh=100

dwqs_list_rh <- gwqsrh(bmi_cat ~ pwqs + nwqs + pad680 + tot_act_cat + riagendr + ridageyr +

race3 + indfmpir + dmdeduc2 + smoke_status + cycle,

mix_name = nutrients, data = tiwqs_data, na.action = na.omit, q = 10,

rh = 3, validation = 0.6, b = 2, family = binomial,

lambda = 100, seed = 123, plan_strategy = "multisession",

solve_dir_issue = "inverse")

summary(dwqs_list_rh)

dwqs_list_rh$final_weights

#####################

# Simulationn study #

#####################

# Create the simulated data

N <- 500

Nnutrients <- length(nutrients)

mu <- colMeans(tiwqs_data[,nutrients])

vcovm <- cor(tiwqs_data[,nutrients], method = "spearman")

w <- rbind(melt(dwqs_list_rh$wmat$wmatpos, value.name = "weight", varnames = c("it", "mix_name")) %>% mutate(direction = "pos"),

melt(dwqs_list_rh$wmat$wmatneg, value.name = "weight", varnames = c("it", "mix_name")) %>% mutate(direction = "neg"))

dwqsrh_w <- w %>%

group_by(mix_name, direction) %>%

summarise(weight = median(weight)) %>%

mutate(weight = ifelse(weight<=1/38, 0, weight))

dwqsrh_w$weight[dwqsrh_w$direction=="pos"] <- dwqsrh_w$weight[dwqsrh_w$direction=="pos"]/sum(dwqsrh_w$weight[dwqsrh_w$direction=="pos"])

dwqsrh_w$weight[dwqsrh_w$direction=="neg"] <- dwqsrh_w$weight[dwqsrh_w$direction=="neg"]/sum(dwqsrh_w$weight[dwqsrh_w$direction=="neg"])

mpweights <- dwqsrh_w$weight[dwqsrh_w$direction=="pos"]

mnweights <- dwqsrh_w$weight[dwqsrh_w$direction=="neg"]

set.seed(123)

create_data <- function(i, vcovm){

dataset <- scale(mvrnorm(n = N, mu = mu, Sigma = vcovm))

colnames(dataset) <- paste0("z", 1:Nnutrients)

dataset <- as.data.frame(dataset)

tmp <- apply(dataset, MARGIN = 2, FUN = function(i) cut(i, quantile(i, seq(0,1,0.1)), labels=F, include.lowest=T)-1)

colnames(tmp) <- paste0("z", 1:Nnutrients, "q")

dataset <- cbind(dataset, tmp)

dataset$s_pwqs <- as.numeric(as.matrix(dataset[, paste0("z", 1:Nnutrients, "q")]) %*% as.vector(mpweights))

dataset$s_nwqs <- as.numeric(as.matrix(dataset[, paste0("z", 1:Nnutrients, "q")]) %*% as.vector(mnweights))

dataset$y <- rnorm(N, 0.5*dataset$s_pwqs - 0.5*dataset$s_nwqs, 1)

dataset$y2 <- rnorm(N, 0.5*dataset$s_pwqs, 1)

dataset$group <- 0

dataset$group[sample(1:N, round(N*0.6))] <-1

return(dataset)

}

# 100 simulated datasets with original correlation matrix

data_list <- lapply(1:3, create_data, vcovm = vcovm)

# 100 simulated datasets with halved correlation matrix

vcovm2 <- vcovm*0.5

diag(vcovm2) <- diag(vcovm)

data_list0.5 <- lapply(1:3, create_data, vcovm = vcovm2)

############################### tuning parameter lambda ###############################

set.seed(123)

rh_list <- lapply(1:3, function(i) sample(0:nrow(data_list[[1]]), size = round(0.6*nrow(data_list[[1]]))))

plan("multisession")

dwqs_list <- future_lapply(data_list, function(i){

tmp <- lapply(c(0, 1, 10, 100, 1000, 10000), function(j){

tmp2 <- gwqsrh(y ~ pwqs + nwqs, mix_name = paste0("z", 1:38), data = i, q = 10,

validation = NULL, rh = rh_list, b = 2, family = gaussian, seed = 123,

valid_var = "group", lambda = j, signal = "t3")

tmp3 <- summary(tmp2)

tmpmat <- cbind(l = j, aic = tmp3$aic, tmp3$coefficients)

tmpw <- tmp2$final_weights

tmpw$l <- j

out <- list(resmat = tmpmat, w = tmpw)

return(out)

})

resmat <- do.call("rbind", lapply(tmp, function(i) i$resmat))

w <- do.call("rbind", lapply(tmp, function(i) i$w))

out2 <- list(resmat = resmat, w = w)

return(out2)

}, future.seed = FALSE)

# AIC vs lambda

reslist <- lapply(dwqs_list, function(i) as.data.frame(i$resmat))

reslist <- lapply(1:length(reslist), function(i){

reslist[[i]]$it <- i

reslist[[i]]$vars <- rownames(reslist[[i]])

rownames(reslist[[i]]) <- NULL

return(reslist[[i]])

})

resall <- as.data.frame(do.call("rbind", reslist))

resall_bars <- resall %>% filter(grepl("^X.Intercep", vars)) %>% group_by(l) %>% summarise(mean_aic = mean(aic), ll_aic = mean(aic)-sd(aic), ul_aic = mean(aic)+sd(aic))

resall_bars

# bias vs lambda

bdata <- resall %>% filter(!grepl("^X.Intercep", vars)) %>%

mutate(vars = factor(ifelse(grepl("^pwqs", vars), "pwqs", "nwqs"), levels = c("pwqs", "nwqs"),

labels = c("Positive direction", "Negative direction")),

beta = ifelse(vars == "Positive direction", Estimate-0.5, Estimate+0.5),

l = factor(l)) %>%

group_by(vars, l) %>%

summarise(median_beta = median(beta), Q1_beta = quantile(beta, probs = 0.25), Q3_beta = quantile(beta, probs = 0.75))

bdata

# Sensitivity and specificity in identifying the true weights by different lambda

wlist <- lapply(dwqs_list, function(i) as.data.frame(i$w))

wlist <- lapply(1:length(wlist), function(i){

wlist[[i]] <- melt(wlist[[i]] %>% dplyr::select(mix_name, l, `Estimate pos`, `Estimate neg`), id.vars = c("mix_name", "l"), value.name = "mean_weight", variable.name = "vars") %>%

mutate(vars = ifelse(vars == "Estimate pos", "pwqs", "nwqs"),

it = i)

return(wlist[[i]])

})

wdata <- as.data.frame(do.call("rbind", wlist))

wdata_se_pos <- wdata %>%

filter(vars == "pwqs") %>%

mutate(correct = mix_name %in% paste0("z", which(mpweights>0)) & mean_weight > 1/38) %>%

group_by(l, it) %>%

summarise(se = sum(correct)/sum(mpweights>0)) %>%

ungroup() %>%

group_by(l) %>%

summarise(se_avg = mean(se))

wdata_sp_pos <- wdata %>%

filter(vars == "pwqs") %>%

mutate(correct = !(mix_name %in% paste0("z", which(mpweights>0))) & mean_weight < 1/38) %>%

group_by(l, it) %>%

summarise(sp = sum(correct)/sum(mpweights==0)) %>%

ungroup() %>%

group_by(l) %>%

summarise(sp_avg = mean(sp))

wdata_se_sp_pos <- left_join(wdata_se_pos, wdata_sp_pos)

wdata_se_sp_pos

wdata_se_neg <- wdata %>%

filter(vars == "nwqs") %>%

mutate(correct = mix_name %in% paste0("z", which(mnweights>0)) & mean_weight > 1/38) %>%

group_by(l, it) %>%

summarise(se = sum(correct)/sum(mnweights>0)) %>%

ungroup() %>%

group_by(l) %>%

summarise(se_avg = mean(se))

wdata_sp_neg <- wdata %>%

filter(vars == "nwqs") %>%

mutate(correct = !(mix_name %in% paste0("z", which(mnweights>0))) & mean_weight < 1/38) %>%

group_by(l, it) %>%

summarise(sp = sum(correct)/sum(mnweights==0)) %>%

ungroup() %>%

group_by(l) %>%

summarise(sp_avg = mean(sp))

wdata_se_sp_neg <- left_join(wdata_se_neg, wdata_sp_neg)

wdata_se_sp_neg

############################### Original correlation matrix ###############################

plan("multisession")

dwqs_list <- future_lapply(data_list, function(i){

tmp1p <- gwqsrh(y ~ wqs, mix_name = paste0("z", 1:38), data = i, q = 10, validation = NULL,

b = 3, rh = rh_list, b1_pos = T, b1_constr = T, family = gaussian, seed = 123,

valid_var = "group")

tmp1n <- gwqsrh(y ~ wqs, mix_name = paste0("z", 1:38), data = i, q = 10, validation = NULL,

b = 3, rh = rh_list, b1_pos = F, b1_constr = T, family = gaussian, seed = 123,

valid_var = "group")

tmp1pcoef <- data.frame(wqs = tmp1p$fit$coefficients[2,1], method = "method 1", vars = "pwqs")

tmp1pw <- tmp1p$final_weights[,1:2]

names(tmp1pw) <- c("mix_name", "mean_weight")

tmp1pw$method <- "method 1"

tmp1pw$vars <- "pwqs"

tmp1ncoef <- data.frame(wqs = tmp1n$fit$coefficients[2,1], method = "method 1", vars = "nwqs")

tmp1nw <- tmp1n$final_weights[,1:2]

names(tmp1nw) <- c("mix_name", "mean_weight")

tmp1nw$method <- "method 1"

tmp1nw$vars <- "nwqs"

i$pwqs <- i$nwqs <- NA

i$pwqs <- tmp1p$wqs

i$nwqs <- tmp1n$wqs

tmp2 <- lm(y ~ pwqs + nwqs, i)

tmp2coef <- data.frame(wqs = tmp2$coefficients[2:3], method = "method 2",

vars = c("pwqs", "nwqs"))

i$pwqs <- i$nwqs <- NULL

tmp3 <- gwqsrh(y ~ pwqs + nwqs, mix_name = paste0("z", 1:38), data = i, q = 10,

validation = NULL, b = 3, rh = rh_list, family = gaussian, seed = 123,

valid_var = "group", lambda = 100, signal = "t3")

tmp3coef <- data.frame(wqs = tmp3$fit$coefficients[2:3,1], method = "method 4",

vars = c("pwqs", "nwqs"))

tmp3w <- tmp3$final_weights[,c(1,2,5)] %>% melt(variable.name = "vars", value.name = "mean_weight")

tmp3w$method <- "method 4"

tmp3w$vars <- ifelse(tmp3w$vars == "Estimate pos", "pwqs", "nwqs")

tmp4 <- qgcomp.noboot(y ~ ., data = i[, c(paste0("z", 1:38), "y")], q = 10)

tmp4coef <- data.frame(wqs = c(tmp4$pos.psi, tmp4$neg.psi), method = "method 3", vars = c("pos.psi", "neg.psi"))

tmpcoef <- rbind(tmp1pcoef, tmp1ncoef, tmp2coef, tmp4coef, tmp3coef)

tmpw <- rbind(tmp1pw, tmp1nw, tmp3w)

rownames(tmpcoef) <- rownames(tmpw) <- NULL

out <- list(resmat = tmpcoef, w = tmpw)

return(out)

}, future.seed = FALSE)

resmat <- do.call("rbind", lapply(dwqs_list, function(i) i$resmat)) %>%

group_by(vars, method) %>%

summarise(median_bias = median(ifelse(vars %in% c("pwqs", "pos.psi"), wqs-0.5, wqs+0.5)),

Q1_bias = quantile(ifelse(vars %in% c("pwqs", "pos.psi"), wqs-0.5, wqs+0.5), probs = 0.25),

Q3_bias = quantile(ifelse(vars %in% c("pwqs", "pos.psi"), wqs-0.5, wqs+0.5), probs = 0.75))

resmat

w <- do.call("rbind", lapply(dwqs_list, function(i) i$w))

wdata_se_pos <- w %>%

filter(vars == "pwqs") %>%

mutate(correct = mix_name %in% paste0("z", which(mpweights>0)) & mean_weight > 1/38,

it = rep(1:length(data_list), each = 38*2)) %>%

group_by(method, it) %>%

summarise(se = sum(correct)/sum(mpweights>0)) %>%

ungroup() %>%

group_by(method) %>%

summarise(se_avg = mean(se))

wdata_sp_pos <- w %>%

filter(vars == "pwqs") %>%

mutate(correct = !(mix_name %in% paste0("z", which(mpweights>0))) & mean_weight < 1/38,

it = rep(1:length(data_list), each = 38*2)) %>%

group_by(method, it) %>%

summarise(sp = sum(correct)/sum(mpweights==0)) %>%

ungroup() %>%

group_by(method) %>%

summarise(sp_avg = mean(sp))

wdata_se_sp_pos <- left_join(wdata_se_pos, wdata_sp_pos)

wdata_se_sp_pos

wdata_se_neg <- w %>%

filter(vars == "nwqs") %>%

mutate(correct = mix_name %in% paste0("z", which(mnweights>0)) & mean_weight > 1/38,

it = rep(1:length(data_list), each = 38*2)) %>%

group_by(method, it) %>%

summarise(se = sum(correct)/sum(mnweights>0)) %>%

ungroup() %>%

group_by(method) %>%

summarise(se_avg = mean(se))

wdata_sp_neg <- w %>%

filter(vars == "nwqs") %>%

mutate(correct = !(mix_name %in% paste0("z", which(mnweights>0))) & mean_weight < 1/38,

it = rep(1:length(data_list), each = 38*2)) %>%

group_by(method, it) %>%

summarise(sp = sum(correct)/sum(mnweights==0)) %>%

ungroup() %>%

group_by(method) %>%

summarise(sp_avg = mean(sp))

wdata_se_sp_neg <- left_join(wdata_se_neg, wdata_sp_neg)

wdata_se_sp_neg

############################### Halving original correlation matrix ###############################

plan("multisession")

dwqs_list <- future_lapply(data_list0.5, function(i){

tmp1p <- gwqsrh(y ~ wqs, mix_name = paste0("z", 1:38), data = i, q = 10, validation = NULL,

b = 3, rh = rh_list, b1_pos = T, b1_constr = T, family = gaussian, seed = 123,

valid_var = "group")

tmp1n <- gwqsrh(y ~ wqs, mix_name = paste0("z", 1:38), data = i, q = 10, validation = NULL,

b = 3, rh = rh_list, b1_pos = F, b1_constr = T, family = gaussian, seed = 123,

valid_var = "group")

tmp1pcoef <- data.frame(wqs = tmp1p$fit$coefficients[2,1], method = "method 1", vars = "pwqs")

tmp1pw <- tmp1p$final_weights[,1:2]

names(tmp1pw) <- c("mix_name", "mean_weight")

tmp1pw$method <- "method 1"

tmp1pw$vars <- "pwqs"

tmp1ncoef <- data.frame(wqs = tmp1n$fit$coefficients[2,1], method = "method 1", vars = "nwqs")

tmp1nw <- tmp1n$final_weights[,1:2]

names(tmp1nw) <- c("mix_name", "mean_weight")

tmp1nw$method <- "method 1"

tmp1nw$vars <- "nwqs"

i$pwqs <- i$nwqs <- NA

i$pwqs <- tmp1p$wqs

i$nwqs <- tmp1n$wqs

tmp2 <- lm(y ~ pwqs + nwqs, i)

tmp2coef <- data.frame(wqs = tmp2$coefficients[2:3], method = "method 2",

vars = c("pwqs", "nwqs"))

i$pwqs <- i$nwqs <- NULL

tmp3 <- gwqsrh(y ~ pwqs + nwqs, mix_name = paste0("z", 1:38), data = i, q = 10,

validation = NULL, b = 3, rh = rh_list, family = gaussian, seed = 123,

valid_var = "group", lambda = 100, signal = "t3")

tmp3coef <- data.frame(wqs = tmp3$fit$coefficients[2:3,1], method = "method 4",

vars = c("pwqs", "nwqs"))

tmp3w <- tmp3$final_weights[,c(1,2,5)] %>% melt(variable.name = "vars", value.name = "mean_weight")

tmp3w$method <- "method 4"

tmp3w$vars <- ifelse(tmp3w$vars == "Estimate pos", "pwqs", "nwqs")

tmp4 <- qgcomp.noboot(y ~ ., data = i[, c(paste0("z", 1:38), "y")], q = 10)

tmp4coef <- data.frame(wqs = c(tmp4$pos.psi, tmp4$neg.psi), method = "method 3", vars = c("pos.psi", "neg.psi"))

tmpcoef <- rbind(tmp1pcoef, tmp1ncoef, tmp2coef, tmp4coef, tmp3coef)

tmpw <- rbind(tmp1pw, tmp1nw, tmp3w)

rownames(tmpcoef) <- rownames(tmpw) <- NULL

out <- list(resmat = tmpcoef, w = tmpw)

return(out)

}, future.seed = FALSE)

resmat <- do.call("rbind", lapply(dwqs_list, function(i) i$resmat)) %>%

group_by(vars, method) %>%

summarise(median_bias = median(ifelse(vars %in% c("pwqs", "pos.psi"), wqs-0.5, wqs+0.5)),

Q1_bias = quantile(ifelse(vars %in% c("pwqs", "pos.psi"), wqs-0.5, wqs+0.5), probs = 0.25),

Q3_bias = quantile(ifelse(vars %in% c("pwqs", "pos.psi"), wqs-0.5, wqs+0.5), probs = 0.75))

resmat

w <- do.call("rbind", lapply(dwqs_list, function(i) i$w))

wdata_se_pos <- w %>%

filter(vars == "pwqs") %>%

mutate(correct = mix_name %in% paste0("z", which(mpweights>0)) & mean_weight > 1/38,

it = rep(1:length(data_list), each = 38*2)) %>%

group_by(method, it) %>%

summarise(se = sum(correct)/sum(mpweights>0)) %>%

ungroup() %>%

group_by(method) %>%

summarise(se_avg = mean(se))

wdata_sp_pos <- w %>%

filter(vars == "pwqs") %>%

mutate(correct = !(mix_name %in% paste0("z", which(mpweights>0))) & mean_weight < 1/38,

it = rep(1:length(data_list), each = 38*2)) %>%

group_by(method, it) %>%

summarise(sp = sum(correct)/sum(mpweights==0)) %>%

ungroup() %>%

group_by(method) %>%

summarise(sp_avg = mean(sp))

wdata_se_sp_pos <- left_join(wdata_se_pos, wdata_sp_pos)

wdata_se_sp_pos

wdata_se_neg <- w %>%

filter(vars == "nwqs") %>%

mutate(correct = mix_name %in% paste0("z", which(mnweights>0)) & mean_weight > 1/38,

it = rep(1:length(data_list), each = 38*2)) %>%

group_by(method, it) %>%

summarise(se = sum(correct)/sum(mnweights>0)) %>%

ungroup() %>%

group_by(method) %>%

summarise(se_avg = mean(se))

wdata_sp_neg <- w %>%

filter(vars == "nwqs") %>%

mutate(correct = !(mix_name %in% paste0("z", which(mnweights>0))) & mean_weight < 1/38,

it = rep(1:length(data_list), each = 38*2)) %>%

group_by(method, it) %>%

summarise(sp = sum(correct)/sum(mnweights==0)) %>%

ungroup() %>%

group_by(method) %>%

summarise(sp_avg = mean(sp))

wdata_se_sp_neg <- left_join(wdata_se_neg, wdata_sp_neg)

wdata_se_sp_neg

############################### Unidirectional association ###############################

plan("multisession")

dwqs_list <- future_lapply(data_list, function(i){

tmp1p <- gwqsrh(y2 ~ wqs, mix_name = paste0("z", 1:38), data = i, q = 10, validation = NULL,

b = 3, rh = rh_list, b1_pos = T, b1_constr = T, family = gaussian, seed = 123,

valid_var = "group")

tmp1n <- gwqsrh(y2 ~ wqs, mix_name = paste0("z", 1:38), data = i, q = 10, validation = NULL,

b = 3, rh = rh_list, b1_pos = F, b1_constr = T, family = gaussian, seed = 123,

valid_var = "group", solve_dir_issue = "average")

tmp3 <- gwqsrh(y2 ~ pwqs + nwqs, mix_name = paste0("z", 1:38), data = i, q = 10, validation = NULL,

b = 3, rh = rh_list, family = gaussian, seed = 123, valid_var = "group",

lambda = 1000)

tmp31d <- gwqsrh(y2 ~ wqs, mix_name = paste0("z", 1:38), data = i, q = 10, validation = NULL,

b = 3, b1_pos = T, family = gaussian, seed = 123, valid_var = "group",

lambda = 1000, rh = rh_list)

tmp1pcoef <- data.frame(wqs = tmp1p$fit$coefficients[2,1], method = "method 1", vars = "pwqs")

tmp1pw <- tmp1p$final_weights[,1:2]

tmp1pw$method <- tmp1pw$method <- "method 1"

tmp1pw$vars <- "pwqs"

tmp1ncoef <- data.frame(wqs = tmp1n$fit$coefficients[2,1], method = "method 1", vars = "nwqs")

tmp1nw <- tmp1n$final_weights[,1:2]

tmp1nw$method <- tmp1nw$method <- "method 1"

tmp1nw$vars <- "nwqs"

i$pwqs <- i$nwqs <- NA

i$pwqs <- tmp1p$wqs

i$nwqs <- tmp1n$wqs

tmp2 <- lm(y2 ~ pwqs + nwqs, i)

tmp2coef <- data.frame(wqs = tmp2$coefficients[2:3], method = "method 2", vars = c("pwqs", "nwqs"))

tmp3coef <- data.frame(wqs = tmp3$fit$coefficients[2:3,1], method = "method 4", vars = c("pwqs", "nwqs"))

tmp3w <- tmp3$final_weights[,c(1,2,5)] %>% melt(variable.name = "vars", value.name = "Estimate")

tmp3w$method <- "method 4"

tmp3w$vars <- ifelse(tmp3w$vars == "Estimate pos", "pwqs", "nwqs")

tmp31dcoef <- data.frame(wqs = tmp31d$fit$coefficients[2,1], method = "method 4 1d", vars = c("pwqs"))

tmp31dw <- tmp31d$final_weights[,c(1,2)]

tmp31dw$method <- "method 4 1d"

tmp31dw$vars <- "pwqs"

tmp4 <- qgcomp.noboot(y2 ~ ., data = i[, c(paste0("z", 1:38), "y2")], q = 10)

tmp4coef <- data.frame(wqs = c(tmp4$pos.psi, tmp4$neg.psi), method = "method 3", vars = c("pwqs", "nwqs"))

tmpcoef <- rbind(tmp1pcoef, tmp1ncoef, tmp2coef, tmp3coef, tmp31dcoef, tmp4coef)

tmpw <- rbind(tmp1pw, tmp1nw, tmp3w, tmp31dw)

rownames(tmpcoef) <- rownames(tmpw) <- NULL

out <- list(resmat = tmpcoef, w = tmpw)

return(out)

}, future.seed = FALSE)

resmat <- do.call("rbind", lapply(dwqs_list, function(i) i$resmat))

w <- do.call("rbind", lapply(dwqs_list, function(i) i$w))

resmat <- do.call("rbind", lapply(dwqs_list, function(i) i$resmat)) %>%

group_by(vars, method) %>%

summarise(median_bias = median(if_else(vars == "pwqs", wqs-0.5, wqs)),

Q1_bias = quantile(if_else(vars == "pwqs", wqs-0.5, wqs), probs = 0.25),

Q3_bias = quantile(if_else(vars == "pwqs", wqs-0.5, wqs), probs = 0.75))

resmat

w <- do.call("rbind", lapply(dwqs_list, function(i) i$w))

wdata_se_pos <- w %>%

filter(vars == "pwqs") %>%

mutate(correct = mix_name %in% paste0("z", which(mpweights>0)) & Estimate > 1/38,

it = rep(1:length(data_list), each = 38*3)) %>%

group_by(method, it) %>%

summarise(se = sum(correct)/sum(mpweights>0)) %>%

ungroup() %>%

group_by(method) %>%

summarise(se_avg = mean(se))

wdata_sp_pos <- w %>%

filter(vars == "pwqs") %>%

mutate(correct = !(mix_name %in% paste0("z", which(mpweights>0))) & Estimate < 1/38,

it = rep(1:length(data_list), each = 38*3)) %>%

group_by(method, it) %>%

summarise(sp = sum(correct)/sum(mpweights==0)) %>%

ungroup() %>%

group_by(method) %>%

summarise(sp_avg = mean(sp))

wdata_se_sp_pos <- left_join(wdata_se_pos, wdata_sp_pos)

wdata_se_sp_pos
